# Dosimetric comparison of three-dimensional conformal radiotherapy versus volumetric-arc radiotherapy in cervical cancer treatment: applying the central-shielding principle to modern technology

**DOI:** 10.1093/jrr/rry054

**Published:** 2018-07-21

**Authors:** Tomoaki Tamaki, Ryuta Hirai, Mitsunobu Igari, Yu Kumazaki, Shin-ei Noda, Yoshiyuki Suzuki, Shingo Kato

**Affiliations:** 1 Department of Radiation Oncology, Fukushima Medical University, 1 Hikarigaoka, Fukushima, Fukushima, Japan; 2 Department of Radiation Oncology, Saitama Medical University International Medical Center, 1397–1 Yamane, Hidaka, Saitama, Japan

**Keywords:** cervical cancer, central shielding, intensity-modulated radiation therapy, volumetric-arc radiotherapy

## Abstract

This study evaluated the feasibility of applying volumetric-arc radiotherapy (VMAT) in standard curative radiotherapy for non-bulky cervical cancer using the central-shielding principle. Whole-pelvis irradiation of 20 Gy and central-shielding pelvis irradiation of 30 Gy, both in 2 Gy fractions, were created using 3D conformal radiotherapy (3DCRT) with a standard midline block or VMAT. Composite dose distributions and DVH parameters were compared first in a simple phantom model and then in 10 clinical cases of Stage I–II cervical cancer. Whole-pelvis clinical target volumes (CTVs) were created from published guidelines for primary disease and lymph node regions, and CTVs for central-shielding irradiation were created by subtracting uterus corpus and 4 cm-wide regions centered at the cervical canal and vagina. In a phantom model, VMAT provided adequate dose coverage to the PTVs without excessive doses to the rectum or bladder compared with the 3DCRT plan. In the clinical cases, VMAT plans resulted in slightly but significantly better coverage of PTVs. The DVH parameters for the rectum and bladder were equivalent or lower for VMAT plans compared with the 3DCRT plans. In the bowel, V_30Gy_, V_40Gy_, and V_50Gy_ were significantly lower in VMAT plans compared with in the 3DCRT plans (47.6% vs 61.0%, 29.8% vs 56.2% and 6.8% vs 21.1%, respectively). Based on these results, VMAT may be used in external-beam radiotherapy for early-stage cervical cancer by adopting the principle of central-shielding pelvis irradiation. Furthermore, VMAT is likely to reduce doses to the small bowel and may reduce gastrointestinal toxicities for these patients.

## INTRODUCTION

Radiotherapy is a major part of the treatment for cervical cancer. Advancements in radiotherapy, both in external-beam radiotherapy (EBRT) and brachytherapy, have led to improved clinical outcomes in terms of disease control as well as in therapy-related toxicities. In Japan, pelvic irradiation with ‘central shielding’ (CS) has been used as a part of standard radiotherapy for cervical cancer since 1980 [1]. CS is generally achieved by placing a rectangular midline block or equivalent shielding with multileaf collimators in the AP–PA fields. The aim of CS is to lower the radiation dose to the bladder and rectum, while providing the necessary dose to the pelvis, specifically to the pelvic lymph node regions and parametrial tissues. The use of CS is one of the reasons for the low incidence of late toxicities in the rectum and bladder [2–4]. This treatment strategy results in relatively low radiation doses (20 Gy/10 fractions to 30 Gy/15 fractions) being delivered to the center of the primary disease by EBRT in relation to the total EBRT pelvic dose (50 Gy/25 fractions). In addition, this strategy allows intracavity brachytherapy to deliver an adequate radiation dose to the primary tumor while reducing doses to the rectum and bladder from EBRT [5, 6].

Intensity-modulated radiation therapy (IMRT) is used in EBRT for curative radiotherapy [7–10] and post-operative radiotherapy [11–14] for cervical cancer. The use of IMRT in curative radiotherapy results in superior dosimetry in the small bowel [9], lower acute gastrointestinal (GI) toxicities [7, 9, 10], and lower chronic or late GI toxicities [8, 10]. In post-operative pelvic chemoradiotherapy, the use of IMRT results in lower chronic GI toxicities [11, 12], lower incidence of bowel obstruction [13], and higher cost-effectiveness due to reduced overall late toxicities [14] compared with conventional 3-dimensional conformal radiotherapy (3DCRT). Recently developed, ‘volumetric-arc radiotherapy’ (VMAT) is an advanced form of IMRT that provides intensity-modulated beams in continuous arcs and has been shown to produce similar or superior dose distributions to static-port IMRT, with a shorter treatment time [15, 16]. The use of static-port IMRT and VMAT has been mostly limited to whole-pelvis irradiation, and there is only a limited number of reports on the use of IMRT to replace a special type of central-shielding called the step-wedge technique [17].

In standard radiotherapy for cervical cancer in Japan, the use of CS with the 3DCRT technique is recommended, even though more sophisticated EBRT technologies are available. This study aimed to assess how cervical cancer patients could receive the benefits of advanced EBRT techniques such as VMAT within the framework of the current standard radiotherapy practice that takes advantage of CS pelvis irradiation. If VMAT can be used for EBRT with the same treatment philosophy, treatment-related toxicities may be reduced not only in the bladder and rectum but also in the non-rectum GI system. In this study, we first conducted a study with a phantom model to verify whether the application of IMRT or VMAT with the CS principle is feasible without causing a significant increase in the radiation dose to the rectum and bladder. Next, we conducted a planning dosimetric study of 10 Clinical Stage I–II cervical cancer patients to compare the applications of the 3DCRT and VMAT in EBRT for cervical cancer.

## MATERIALS AND METHODS

### Phantom model planning

**Fig. 1.**
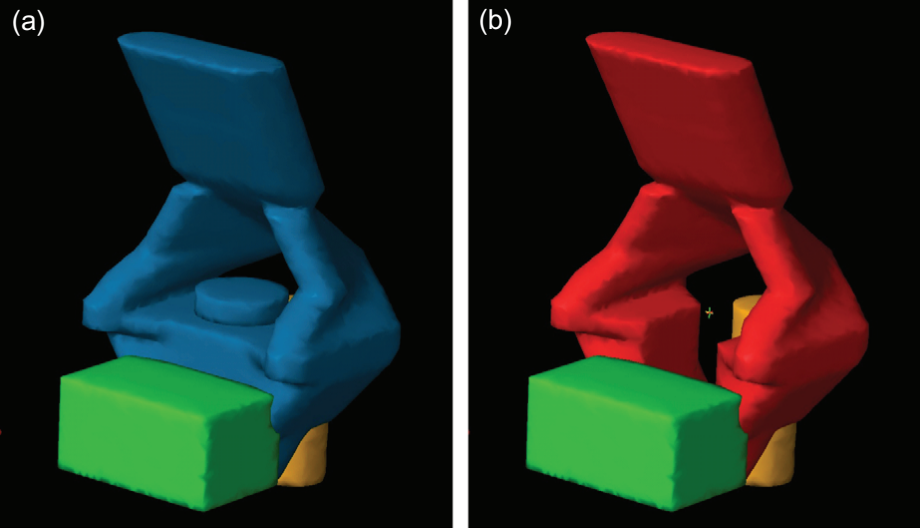
Planning target volumes (PTVs) and organs at risk used for the phantom study: (a) the PTV for whole-pelvis irradiation (PTV-WP_phantom_) is shown in blue, with the bladder (green) and rectum (yellow); and (b) the PTV for pelvis irradiation with central-shielding (PTV-CS_phantom_) is shown in red, with the bladder_phantom_ (green) and rectum_phantom_ (yellow).

Based on the anatomy of the female pelvis, a simple virtual model defining the clinical target volume for whole-pelvis irradiation (CTV-WP_phantom_), including the pelvic lymph node region, parametrial tissue, uterus, and upper vagina, was created on a dosimetric phantom for the initial treatment-planning study (see Supplementary Fig. 1). For pelvis irradiation with the CS principle, a 4 cm width at the central part of the pelvis (uterus corpus, upper vagina, and some parametrial tissue of the phantom) was removed from the CTV-WP_phantom_. This volume was termed the CTV-CS_phantom_ (see Supplementary Fig. 2). Simple volumes for the bladder and rectum were also created. The bladder was defined as an 8 cm (right-left) × 5 cm (anterior-posterior) × 4 cm (cranial-caudal) box (bladder_phantom_) situated 0.5 cm anterior to the anterior border of the central part of the CTV-WP_phantom_. The volume of the box that overlapped with the CTV-WP was deleted from the bladder_phantom_. The rectum was defined as a cylinder with a height of 8 cm and a diameter of 4 cm (rectum_phantom_) situated 0.5 cm posterior to the central part of the CTV-WP_phantom_. PTVs were created from the CTV-WP_phantom_ and CTV-CS_phantom_ with 5 mm margins and termed the PTV-WP_phantom_ and PTV-CS_phantom_, respectively (Fig. 1). The detailed axial images of the virtual phantoms are shown in Supplementary Figs 1 and 2.

### Phantom model planning: 3DCRT

**Fig. 2.**
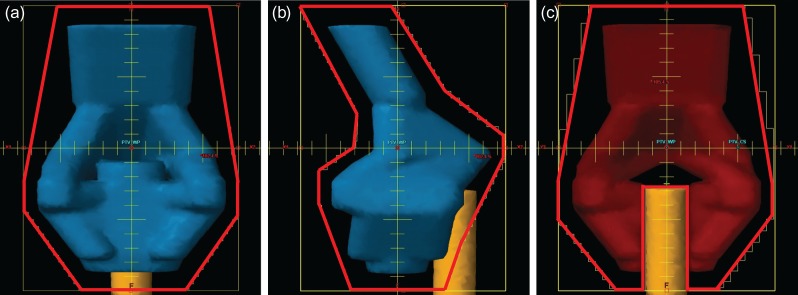
Irradiation fields of the phantom study (shown by red lines): (a) anterior–posterior field of whole-pelvis irradiation; (b) left–right field of whole-pelvis irradiation; and (c) anterior–posterior field of pelvis irradiation with central shielding. The width of the midline block in (c) was set as 3 cm. The PTV-WP_phantom_, PTV-CS_phantom_, and rectum_phantom_ are shown in the figure.

For whole-pelvis irradiation of 20 Gy/10 fractions, a four-field box technique with 10 MV X-ray beams was used so that the 95% isodose line mostly covered the PTV-WP_phantom_ and simulated traditional whole-pelvis irradiation (Fig. 2a and b). For CS pelvis irradiation of 30 Gy/15 fractions, AP–PA fields were created with a 3 cm wide CS block inserted in the center from the caudal side such that it did not compromise the dose coverage of the presacral lymph node region (Fig. 2c).

### Treatment-planning computed tomography and software

For the treatment planning, Eclipse version 11 (Varian Medical Systems, Inc., California, U.S.A.) was used. Treatment planning computed tomography (CT) was obtained with a slice thickness of 2.5 mm, and the grid size for dose calculation was 2.5 mm. An analytical anisotropic algorithm (AAA, version 11.0.31) was used.

### Phantom model planning: static-port IMRT and VMAT

The same PTVs were used for the static-port IMRT plans and VMAT plans. Whole-pelvis irradiation of 20 Gy/10 fractions and CS pelvis irradiation of 30 Gy/15 fractions were created as two-step methods similar to the 3DCRT planning. For static-port IMRT, 7 static ports at 0, 55, 105, 155, 205, 255 and 305 degrees with 10 MV X-ray beams were used. For VMAT, double arcs with 10 MV X-ray beams were used. The planning aim was to cover 50% of the PTV-WP_phantom_ or PTV-CS_phantom_ with the prescribed doses. The planning aims for normal organs for total EBRT were as follows: for the rectum_phantom_, volume receiving 40 Gy (V_40Gy_) < 85%, volume receiving 50 Gy (V_50Gy_) < 40%, maximum dose (D_max_) < 110%; for the bladder_phantom_, V_30Gy_ < 40%; and for the whole body, D_max_ < 115%.

### Actual clinical cases and delineation of targets/organs at risk

Ten patients who received radiotherapy for cervical cancer Stage I–IIB were selected for this planning study. The patients’ characteristics are provided in Supplementary Table 1. This study was approved by the Institutional Review Board of Saitama Medical University International Medical Center. The CTVs for primary disease of the uterine cervix (CTV-PD) and the pelvic lymph node (CTV-LN) were defined based on previously published guidelines [18, 19]. The summed volume of CTV-PD plus CTV-LN was defined as the CTV for whole-pelvis irradiation (CTV-WP). As the clinical target volume of pelvis irradiation with CS (CTV-CS), the uterus corpus and the 4 cm-wide region centered at the cervical canal and the center of vagina, not at the center of the pelvic bony structure, were subtracted from the CTV-WP. For the PTVs, a 5 mm margin was added to the CTV-WP and CTV-CS (PTV-WP and PTV-CS, respectively). For the uterus and vagina, the anterior–posterior margin was increased to 10 mm. Organs at risk, including the bladder, rectum, femoral heads, and bowel, were delineated. The bowel was defined as a bag-like structure including the small and large intestines minus the rectum. For the evaluation of dose distributions, the anterior rectal wall, which was shielded by the 3DCRT CS fields, was identified by the 3-mm inner extension from the outer rectal wall that falls inside the AP–PA projection of the midline shielding formed by the multileaf collimators.

### 3DCRT planning of clinical cases

For the 3DCRT plans, the radiation fields used in actual clinical treatment were applied with some minor modifications. PTVs were adjusted at the cranial end such that adequate dose coverage was achieved by the field border at the top of the fifth lumber spine. For this study, whole-pelvis irradiation of 20 Gy/10 fractions was created with 10 MV X-ray beams by the four-field box technique used in actual treatment, and CS pelvis irradiation of 30 Gy/15 fractions was created with a 3 cm-wide midline block placed at the caudal portion of the center of the bony pelvis structure. The height of the shielding was adjusted such that it did not compromise the presacral lymph node region.

### VMAT planning of clinical cases

For VMAT planning, double-arc VMAT plans using 10 MV X-ray beams were created for the PTV-WP and PTV-CS. Similar to the phantom study, the VMAT plans were created as two-step methods for PTV-WP (20 Gy/10 fractions) and PTV-CS (30 Gy/15 fractions). The dose prescription was based on PTV D_50%_ values with planning aims as follows: PTV D_95%_ > 92%, PTV D_98%_ > 90% and PTV D_2%_ < 110%. The planning aims for OARs were set for the composite doses of WP and CS plans as follows: for the rectum, volume receiving 40 Gy (V_40Gy_) < 85%, volume receiving 50 Gy (V_50Gy_) < 40%, the maximum dose (D_max_) < 110%; for the bladder, D_max_ < 110%; for the bowel, V_40Gy_ < 40%; for the femoral heads, V_30Gy_ < 40%; and for the whole body, D_max_ < 115%. The VMAT plans were optimized such that the dose to the rectum was minimized [20]: the volume of rectum lying within the 2-cm radius from the center of the cervical canal or vagina was identified, and optimization was performed to lower the dose to this volume [20]. Static-port IMRT planning was not done because variability in the selection of port angles and numbers may significantly influence the analysis of DVH parameters in actual clinical cases.

### Evaluation of dose–volume histogram parameters

To evaluate the treatment plans, volumes irradiated with 20 Gy, 30 Gy, 40 Gy and 50 Gy, (V_20Gy_, V_30Gy_, V_40Gy_ and V_50Gy_), the maximum doses (D_max_) for the bladder, rectum and bowel, the V_30_ and D_max_ of the femoral heads, the D_max_ of the model/patient, and the minimum doses delivered to 98%, 95% and 2% (D_98_, D_95_, and D_2_) of the PTVs (PTV-WP and PTV-CS) were analyzed for the composite plans of whole-pelvis irradiation and pelvis irradiation with CS. The mean doses to the anterior rectal wall (3 mm thickness), which was shielded by the 3DCRT CS fields (CS rectum D_mean_), were also analyzed: *t*-tests were used for statistical analysis of the differences between the treatment modalities, and *P* values <0.05 were considered statistically significant.

## RESULTS

**Fig. 3.**
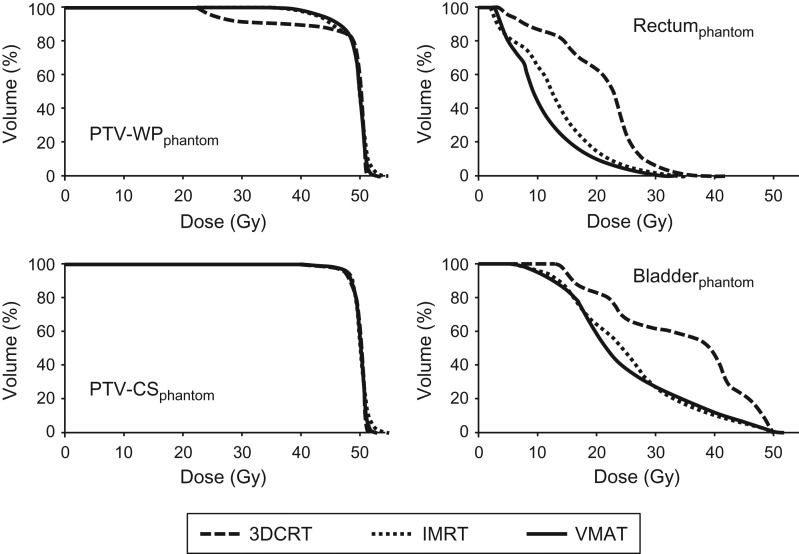
Dose–volume histograms of the PTV-WP_phantom_, PTV-CS_phantom_, rectum_phantom_ and bladder_phantom_ comparing 3DCRT plans, static-port IMRT plans, and VMAT plans in the phantom study.

A planning study on the hypothetical phantom model was first performed to verify the possible increase in radiation dose to the bladder and rectum due to the use of static-port IMRT and VMAT, as these are major OARs in which toxicities can occur. The DVHs and their parameters are shown in Fig. 3 and Table 1. Static-port IMRT plans and VMAT plans showed similarly superior DVH profiles compared with the 3DCRT plans. In the phantom model, V_50Gy_ and D_max_ of the bladder_phantom_ were higher in the IMRT/VMAT plans than in the 3DCRT plan (0.2%/0.7% vs 0% and 103.4%/105.3% vs 99.9%, respectively), while all other parameters in the bladder_phantom_ and rectum_phantom_ were lower in the IMRT/VMAT plans. The coverages of PTV-CS_phantom_ were generally equivalent. The D_98%_ and D_95%_ of PTV-WP_phantom_ were considerately higher in IMRT/VMAT plans compared with in the 3DCRT plans (79.5%/80.6% vs 47.3% and 85.4%/86.6% vs 50.4%, respectively), indicating that the central portion of the PTV-WP_phantom_—including the primary tumor, uterus corpus, and vagina—received a higher dose from the use of static-port IMRT and VMAT compared with the 3DCRT.

**Table 1.** Comparison of DVH parameters in the 3DCRT plans, static-port IMRT plans, and VMAT plans using a phantom model

Volumes	Parameters	3DCRT	IMRT	VMAT
Bladder_phantom_	V_20Gy_	83.6	64.4	59.1
	V_30Gy_	62.5	27.0	27.5
	V_40Gy_	45.5	10.4	12.3
	V_50Gy_	0	0.2	0.7
	D_max_	99.9	103.4	105.3
Rectum_phantom_	V_20Gy_	62.9	15.7	10.4
	V_30Gy_	6.4	1.7	0.8
	V_40Gy_	0.1	0	0
	V_50Gy_	0	0	0
	D_max_	84.9	70.7	66.8
PTV_WP_phantom_	D_98%_	47.3	79.5	80.6
	D_95%_	50.4	85.4	86.6
	D_2%_	102.1	105.3	103.2
PTV_CS_phantom_	D_98%_	90.2	92.0	91.8
	D_95%_	94.5	95.4	95.1
	D_2%_	102.1	105.5	102.1

The parameters are given in percentages (%, for both volume and dose).

**Fig. 4.**
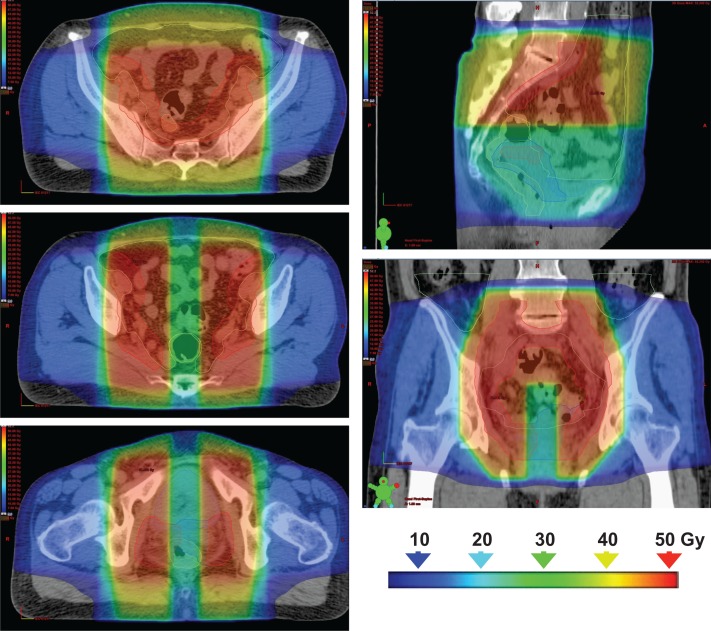
Dose distributions of the 3DCRT plan: the figure shows the composite dose distributions of whole-pelvis irradiation (20 Gy/10 fractions) and pelvis irradiation with central shielding (30 Gy/15 fractions).

**Fig. 5.**
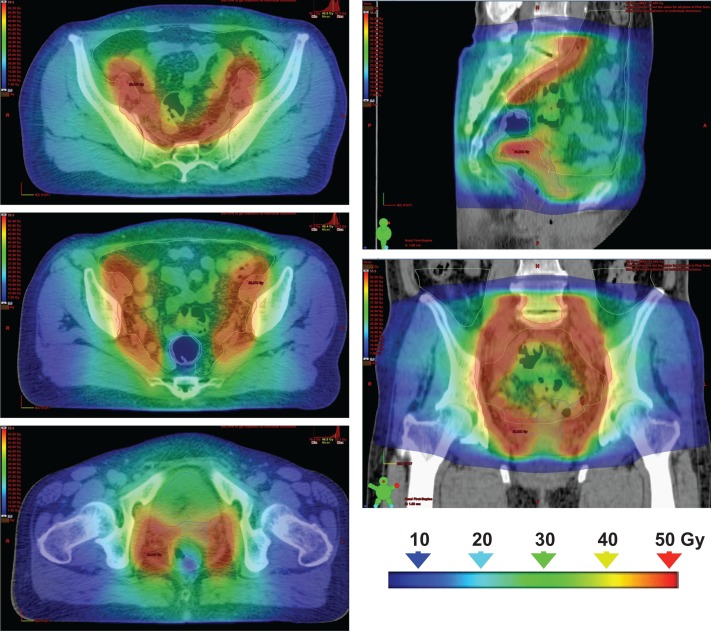
Dose distributions of the VMAT plan (same slices as for the case shown in Fig. 3): the figure shows the composite dose distributions of whole-pelvis irradiation (20 Gy/10 fractions) and pelvis irradiation with the central-shielding philosophy (30 Gy/15 fractions).

**Fig. 6.**
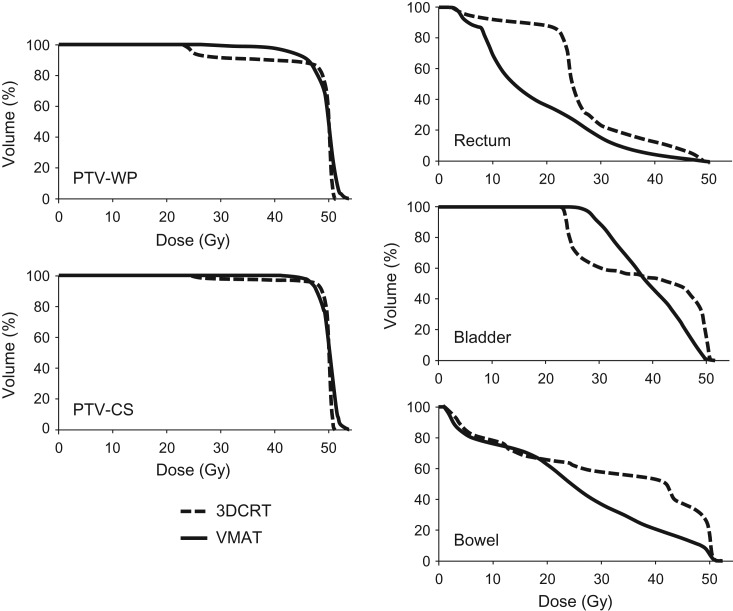
Representative dose–volume histograms of PTV-WP, PTV-CS, rectum, bladder and bowel comparing the 3DCRT plans and VMAT plans in clinical cases.

Table 2 summarizes the DVH parameters of the planning study of 10 clinical cases. Examples of dose distributions by 3DCRT vs VMAT plans are shown in Figs 4 and 5, and representative DVHs of the PTV-WP, PTV-CS, rectum, bladder and bowel are shown in Fig. 6. For the bladder, V_30Gy_ and D_max_ were significantly higher and V_50Gy_ was significantly lower in VMAT plans. For the rectum, V_20Gy_ and V_40Gy_ were significantly lower in VMAT plans, and V_50Gy_ was very low both in the 3DCRT and VMAT plans. The CS rectum D_mean_ did not differ significantly between the 3DCRT and VMAT plans. For the bowel, V_30Gy_, V_40Gy_, and V_50Gy_ were significantly lower and D_max_ was significantly higher in VMAT plans. For the PTV-WP, D_98%_, D_95%_ and D_2%_ were all significantly higher in VMAT plans. For the PTV-CS, D_98%_ and D_2%_ were also significantly higher in VMAT plans.

**Table 2.** Comparison of DVH parameters between the 3DCRT plans and VMAT plans

Volumes	Parameters	3DCRT	VMAT	*P* values
Bladder	V_20Gy_	99.9 ± 0.3	98.5 ± 4.6	0.374
	V_30Gy_	57.0 ± 9.0	81.2 ± 17.2	0.002
	V_40Gy_	48.2 ± 9.9	41.7 ± 10.8	0.136
	V_50Gy_	11.9 ± 12.2	1.6 ± 1.0	0.023
	D_max_	101.4 ± 1.9	104.0 ± 0.9	0.006
Rectum	V_20Gy_	90.4 ± 6.2	46.4 ± 8.1	<0.001
	V_30Gy_	27.8 ± 8.9	21.9 ± 5.6	0.116
	V_40Gy_	18.1 ± 8.7	6.9 ± 2.7	0.001
	V_50Gy_	0.4 ± 0.8	0.0 ± 0.1	0.150
	D_max_	99.5 ± 1.8	99.8 ± 2.6	0.723
CS rectum^a^	D_mean_	49.4 ± 6.0	48.2 ± 6.6	0.597
Bowel	V_20Gy_	68.8 ± 5.9	69.0 ± 5.2	0.886
	V_30Gy_	61.0 ± 5.7	47.6 ± 6.2	<0.001
	V_40Gy_	56.2 ± 5.4	29.8 ± 5.2	<0.001
	V_50Gy_	21.1 ± 6.0	6.8 ± 1.7	<0.001
	D_max_	103.4 ± 1.3	106.9 ± 1.7	<0.001
	V_45Gy_ (cm^3^)	634 ± 217 (cm^3^)	292 ± 68 (cm^3^)	<0.001
Left femur	V_30Gy_	5.5 ± 4.9	2.2 ± 1.5	0.033
	D_max_	96.0 ± 5.0	78.4 ± 9.1	<0.001
Right femur	V_30Gy_	5.2 ± 4.5	2.3 ± 1.3	0.031
	D_max_	93.6 ± 7.7	74.9 ± 4.4	<0.001
Patient	D_max_	103.7 ± 1.0	110.1 ± 1.5	<0.001
PTV_WP	D_98%_	47.5 ± 0.8	69.0 ± 7.3	<0.001
	D_95%_	49.1 ± 1.0	79.7 ± 7.0	<0.001
	D_2%_	102.4 ± 0.9	105.1 ± 0.7	<0.001
PTV_CS	D_98%_	68.8 ± 14.5	90.5 ± 1.4	0.001
	D_95%_	90.6 ± 6.2	93.0 ± 0.9	0.241
	D_2%_	102.5 ± 0.9	105.3 ± 0.7	<0.001

The parameters are given in percentages for both volume and dose (average ± standard deviation). ^a^Anterior wall of the rectum, which was blocked by 3DCRT central shielding.

## DISCUSSION

This planning study showed that the use of IMRT or VMAT resulted in equal or better dose coverage of PTVs without creating excessively high-dose regions in the bladder or rectum, thus maintaining the philosophy of the traditional CS technique. IMRT or VMAT can result in a significantly lower dose being delivered to the bowel, and may offer the advantage of lower GI toxicities compared with conventional 3DCRT.

The purpose of CS is to irradiate the pelvic lymph node regions and parametrial tissues while shielding the bladder, rectum, and primary lesion; thus, by lowering the EBRT dose to the bladder and rectum, this technique enables brachytherapy to provide an effectively high dose to the primary lesion for better control of the disease. CS continues to be the standard in Japan [1–4] and is widely implemented in the USA [21, 22] and in Europe [23]. However, despite its effectiveness, CS has started to be replaced by simple whole-pelvis irradiation in many countries due to the complexity of analyzing and interpreting the doses delivered to the primary tumor. The adoption of whole-pelvis irradiation inevitably increases doses provided by EBRT to the bladder and rectum, resulting in more challenges for clinicians to meet dose constraints during intracavitary brachytherapy treatment. It would be regretful if the advantageous treatment principle of CS was abandoned simply because the irradiation technique may seem outdated. In this study, we attempted to apply VMAT, an advanced technique of EBRT, to cervical cancer using the CS principle to improve EBRT treatment further.

This study showed that reduction of radiation doses to the bladder and rectum can be adequately achieved by IMRT or VMAT. In the phantom model with simple geometry, all of the studied parameters of the bladder and rectum, except the maximum dose to the bladder, were lower in the IMRT or VMAT cases (Table 1 and Fig. 3). Since VMAT provides slightly better dose distributions and has the clinical advantage of shorter treatment times, we support the use of VMAT in clinical cases. In the 10 clinical cases, V_40Gy_ and V_50Gy_ of the bladder and rectum were equivalent or significantly lower in VMAT plans, and the increase in the maximum dose to the bladder was significant, but only by a small amount (<3 Gy, 4.6%). One of our initial concerns was whether the anterior rectal wall, which is conventionally shielded by the midline block in the 3DCRT (CS rectum), would receive a higher dose by VMAT. However, this study showed that the mean dose to this portion of the anterior rectal wall was equivalent between the 3DCRT and VMAT plans (49.4% vs 48.2%, Table 2). Although these data indicate that the use of VMAT maintains equally low doses to the rectum and bladder, the dose distribution should be carefully checked to avoid unintended high-dose regions in organs at risk.

In this study, we showed VMAT could achieve significantly better D_98%_ of the parametrium and pelvic lymph node region (PTV-CS) in clinical cases compared with in 3DCRT, which uses a standardized rectangular CS (D_98%_: 90.5% vs 68.8%, *P* = 0.001). The dose coverage of the PTV-CS was slightly compromised in the 3DCRT plans because the standardized shielding was placed in the center of the bony structure, as practiced clinically, irrespective of the position of the PTV-CS. In previous reports, IMRT has been shown to provide equally good or superior dose coverage of the PTV in whole-pelvis irradiation in curative radiotherapy [7] and post-hysterectomy radiotherapy [11, 12] in cervical cancer patients. VMAT has been shown to provide comparable PTV dose coverage to IMRT in previous studies [15, 16] and in our analysis using a phantom. In this study, analysis of the PTV coverage is more complicated because the PTVs are different in the initial whole-pelvis plans of 20 Gy/10 fractions and CS plans of 30 Gy/15 fractions, and the DVH parameters were analyzed with the composite dose distributions. The dose coverage of PTV-CS and PTV-WP both improved with the use of VMAT, but the difference was more pronounced in PTV-WP than in PTV-CS (Fig. 6). For the 3DCRT plans, PTV-WP can be roughly divided into two volumes based on their dose coverage: the volume irradiated fully without shielding (~50 Gy) and the volume irradiated partially with CS (~20 Gy). (Fig. 6). On the other hand, the central part, which consisted of the primary tumor and uterus, tended to receive a higher dose in VMAT, and this difference was readily seen in the D_95%_ of PTV-WP: 49.1% (~25 Gy) in the 3DCRT and 79.7% (~40 Gy) in VMAT. While the dose to the rectum and bladder can be similarly reduced in VMAT and in the 3DCRT plans, clinicians should be aware the PTV-WP—especially the central region that contains the primary disease—may receive a higher dose in VMAT plans.

This study has envisioned a treatment strategy for relatively early stages of cervical cancer. Toita *et al.* have shown that a radiotherapy regimen with whole-pelvis irradiation of 20 Gy/10 fractions, CS pelvis irradiation of 30 Gy/15 fractions, and 4 fractions of intracavitary brachytherapy resulted in good disease control and little toxicities for non-bulky Stage I–II cervical cancer patients [3]. Because long-term survival is expected for most of these patients, avoiding late toxicities is essential. Our study suggests reduction of late GI toxicities is expected with the use of IMRT or VMAT because they lower doses to the bowel compared with 3DCRT. Mundt *et al.* showed that, compared with conventional 3DCRT radiotherapy, the use of IMRT in pelvis irradiation decreased the incidence of moderate acute GI toxicity in cervical cancer patients (91% vs 60%) [7] and chronic GI toxicity in gynecology patients (50.0% vs 11.1%) [8]. A more recent study by Hassele *et al.* showed that the use of IMRT for cervical cancer patients was estimated to lower the acute and late Grade >3 toxicities to 2% and 7%, respectively [10]. Our results support the hypothesis that IMRT with the principle of using conventional CS is a promising treatment option that can decrease not only toxicities in the rectum and bladder, but also toxicities in the small bowel.

This study showed that, compared with 3DCRT, VMAT achieved significantly better DVH parameters in the bowel, and in particular that it decreased V_30Gy_ (61.0% vs 47.6%), V_40Gy_ (56.2% vs 29.8%) and V_50Gy_ (21.1% vs 6.8%). These DVH parameters were previously reported to correlate with the incidence of GI toxicities [9, 11, 12]. Roeske *et al.* showed that, in gynecology patients receiving whole-pelvis IMRT of 45 Gy/25 fractions, the volume of the small bowel irradiated with a 100% dose was the most significant factor relating to acute GI toxicity [9]. Chen *et al.* showed, in post-hysterectomy cervical cancer patients receiving chemoradiotherapy, that of the total 50.4 Gy/28 fractions, the V_50%_, V_70%_, V_90%_ and V_100%_ and D_mean_ of the small intestine were all lower when IMRT was used, resulting in significantly lower chronic GI toxicities in the IMRT group compared with in the box-field 3DCRT group [11]. Isohashi *et al.* also compared post-hysterectomy patients receiving whole-pelvis irradiation by either 3DCRT or IMRT and reported that the IMRT group showed a lower incidence of acute and chronic GI complications and that the V_40Gy_ and V_45Gy_ of the small bowel loops or bowel bag were predictive of GI complications [12]. In the Quantitative Analyses of Normal Tissue Effects in the Clinic (QUANTEC) guidelines, Kavanagh *et al.* recommended that the volume of the small bowel receiving >45 Gy should be <195 cm^3^ to minimize severe acute toxicities, although correlation with the incidence of late toxicities has not yet been established [24]. These previous reports, as well as our results for bowel V_30Gy_, V_40Gy_ and V_50Gy_, suggest that the use of VMAT may lower the incidence of acute and chronic GI toxicities—especially in the small bowel—in patients with early-stage cervical cancer treated with the CS principle.

When VMAT is considered for cases with primary diseases larger than those expected in this study, the definition of CTV-CS may require some modification. As the HR-CTV becomes larger, CS pelvis irradiation contributes a relatively more substantial radiation dose to the HR-CTV, although the total dose delivered to the HR-CTV inevitably becomes lower [5, 6]. In more advanced cases, the CTV-CS may need to include lateral portions of the central primary disease, not only at the level of parametrial tissue but also at the level of the uterus corpus, depending on the shape and extent of the primary disease. If the cervical canal and the lumen of the uterus can be identified carefully at the time of VMAT planning, composite dose distributions of the EBRT and brachytherapy may be improved further compared with the use of a standardized midline block in the 3DCRT. Thus, the definition of CTV-CS requires further discussion for more advanced cases.

The application of IMRT/VMAT requires careful attention to the allocation of an adequate PTV margin, especially for whole-pelvis irradiation, wherein the movement of the uterus tends to demonstrate the largest inter- and intra-fraction motion [25]. In this study, the PTV margin of 5 mm was added to the CTVs in general, and an increased anterior–posterior margin of 10 mm was added to the uterus and vagina to produce PTV-WP. However, other studies have suggested various values for margins accounting for pelvic organ motion [25]. Chan *et al.* have suggested the internal target margin of the fundus to be as large as 40 mm [26]; conversely, Lim *et al.* have shown that IMRT plans with a 5-mm PTV margin yielded adequate target coverage in most of the patients with cervical cancer in their study [27]. Nevertheless, these studies consistently highlighted the need of each treating institution to establish its own evidence-based margins, often requiring patient-specific PTV margins. Careful measures such as controlling bladder/rectal filling and applying image-guided radiotherapy should be taken [25, 28]. Excessively small margins can result in geographic miss of the target, whereas excessively large margins can compromise the technological advantages of IMRT/VMAT [25].

In conclusion, our study showed that IMRT or VMAT may be used in EBRT for early-stage cervical cancer by adopting the CS principle currently practiced in Japan and in other countries. The application of these technologies is likely to reduce radiation doses to the small bowel and may reduce late GI toxicities for these patients. Further validation of this technique in clinical studies is, thus, warranted.

## Supplementary Material

Supplementary Data

Supplementary Data

Supplementary Data
