# Supplementary material for: Dosimetric comparison of three-dimensional conformal radiotherapy versus volumetric-arc radiotherapy in cervical cancer treatment: applying the central-shielding principle to modern technology
Source: J Radiat Res. 2018 Jul 21;59(5):639–48. doi: 10.1093/jrr/rry054 (PMC6151642; doi:10.1093/jrr/rry054)
Supplement: Supplementary Data [file rry054_supplementary_figure_1_small.pdf]

Supplementary Figure 1: Axial images of the CTV-$WP_{phantom}$ (blue), $bladder_{phantom}$(green), $rectum_{phantom}$ (orange), and PTV-$WP_{phantom}$ (light blue) on a dosimetric phantom. The images are shown with grids of 2 cm and in an interval of 2.5 mm in crainal-caudal direction. The shapes of CTV-$WP_{phantom}$ were simplified based on the average values of CTV dimensions of the 10 clinical cases studied. The measurements were taken at the levels of upper border of the 5th lumber spine, upper border of the first sacral spine, lower border of sacroiliac joint, the widest level of bony pelvis, and the upper border of the pubic symphysis.

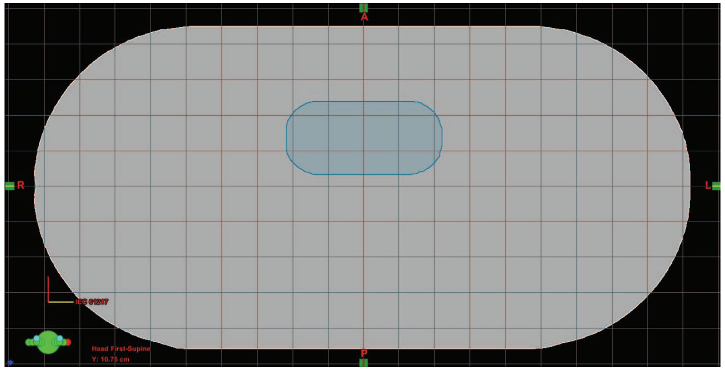
A
R
L
P
IEC 61217
Head First-Supine
Y: 10.75 cm
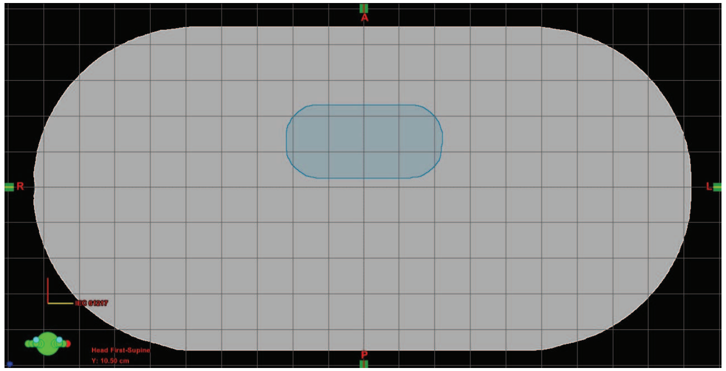
A
R
L
P
IEC 61217
Head First-Supine
Y: 10.50 cm
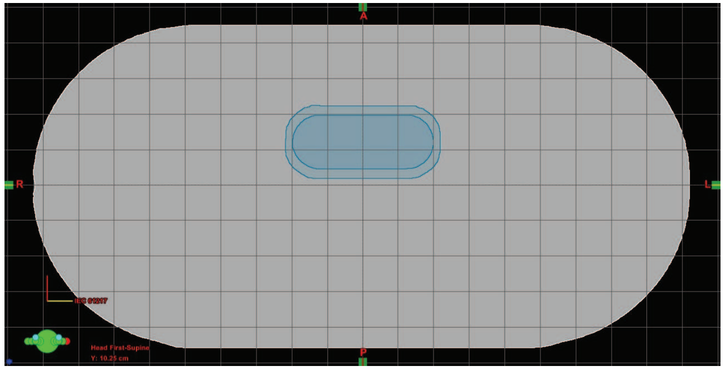
A
R
L
P
IEC 61217
Head First-Supine
Y: 10.25 cm
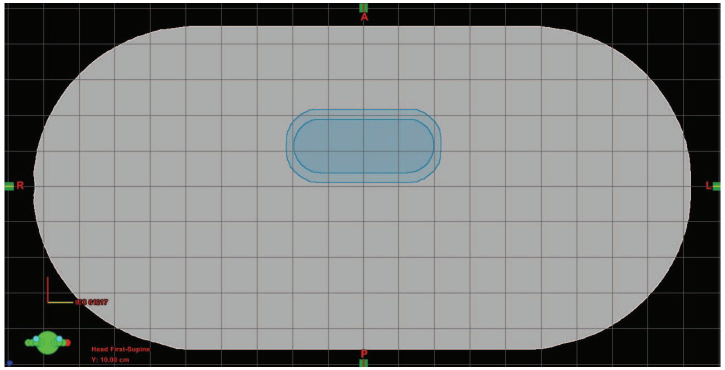
A
R
L
P
IEC 61217
Head First-Supine
Y: 10.00 cm
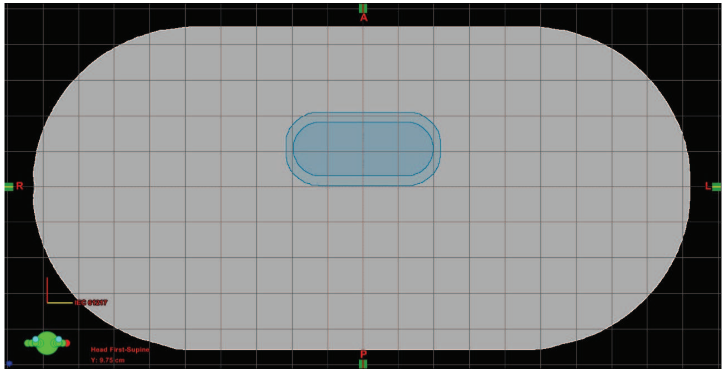
A
R
L
P
IEC 61217
Head First-Supine
Y: 9.75 cm
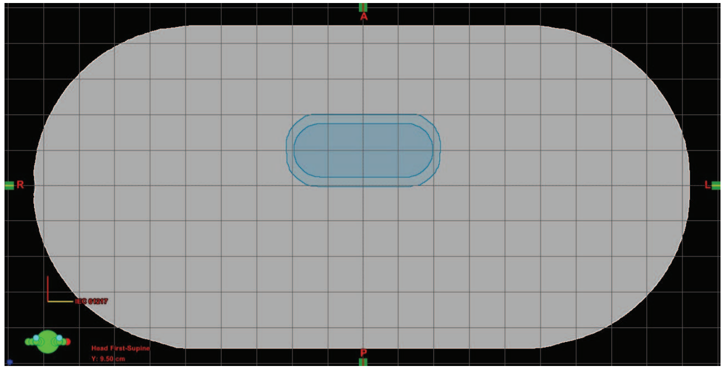
A
R
L
P
IEC 61217
Head First-Supine
Y: 9.50 cm
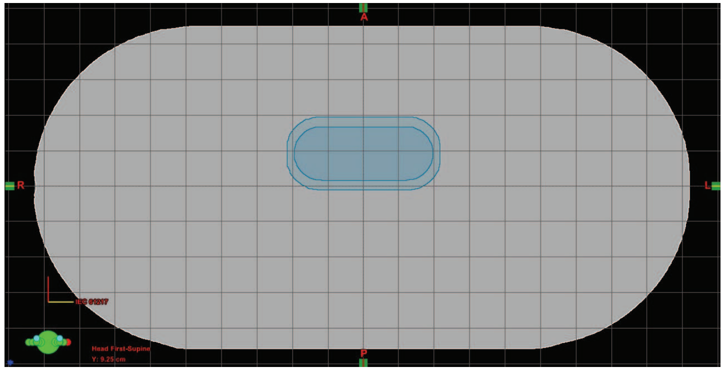
A
R
L
P
IEC 61217
Head First-Supine
Y: 9.25 cm
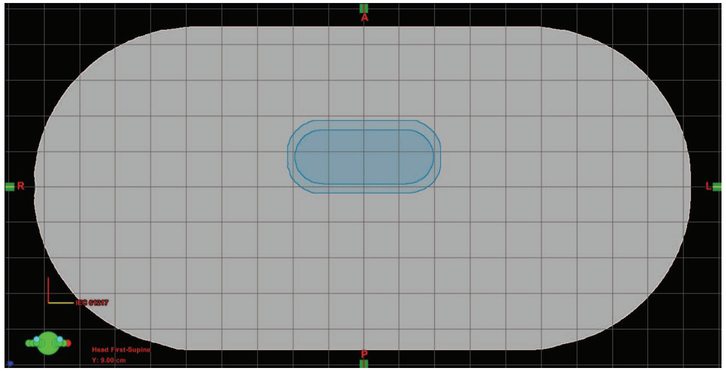
A
R
L
P
IEC 61217
Head First-Supine
Y: 9.00 cm
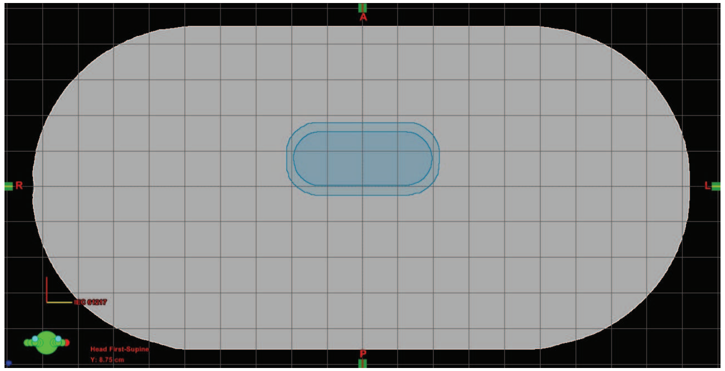
A
R
L
P
IEC 61217
Head First-Supine
Y: 8.75 cm

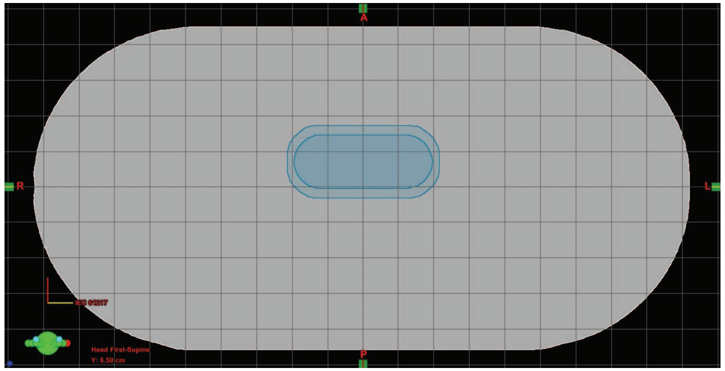
A
R
L
P
IEC 61217
Head First-Supine
Y: 8.50 cm

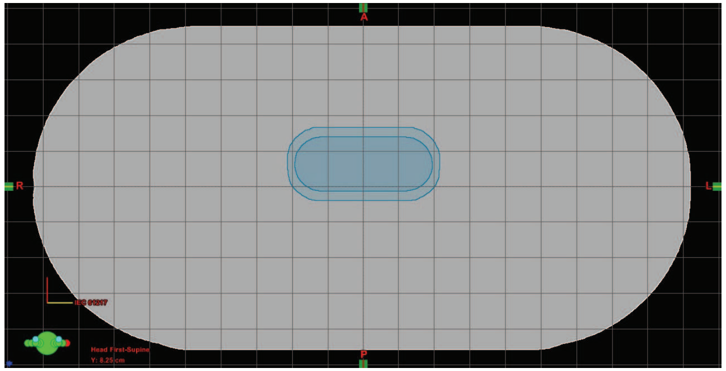
A
R
L
P
IEC 61217
Head First-Supine
Y: 8.25 cm

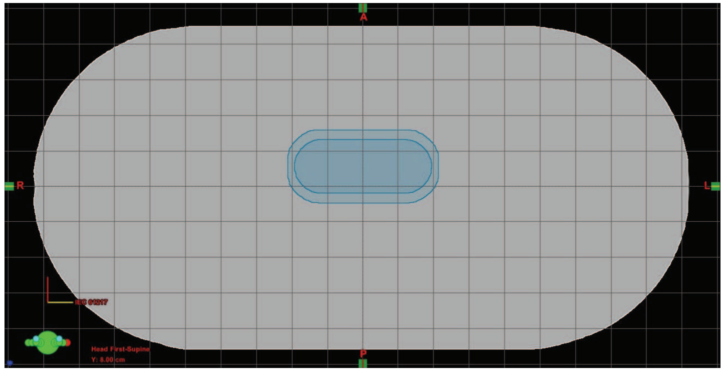
A
R
L
P
IEC 61217
Head First-Supine
Y: 8.00 cm

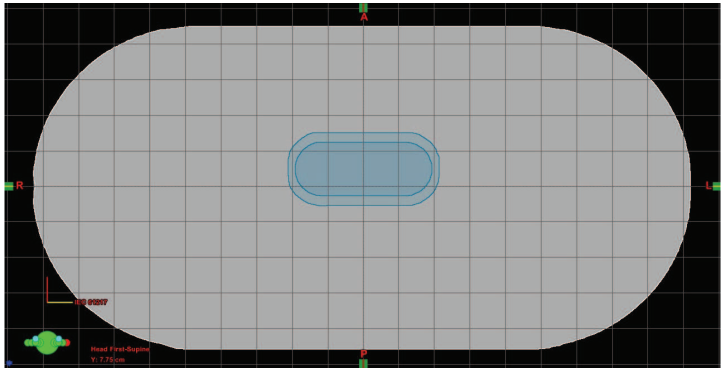
A
R
L
P
IEC 61217
Head First-Supine
Y: 7.75 cm

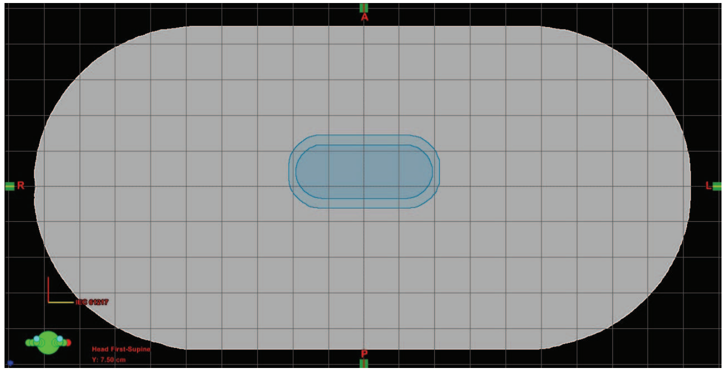
A
R
L
P
IEC 61217
Head First-Supine
Y: 7.50 cm

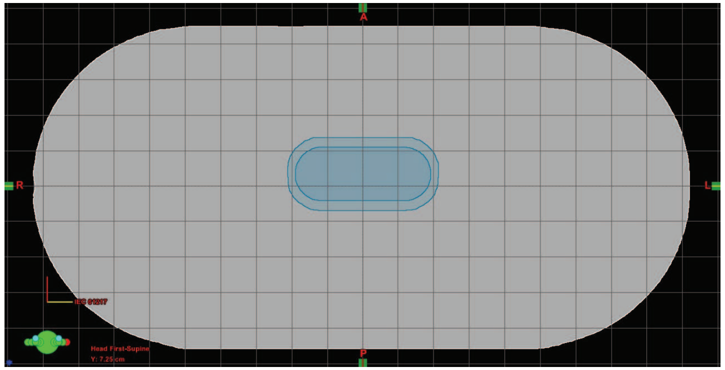
A
R
L
P
IEC 61217
Head First-Supine
Y: 7.25 cm

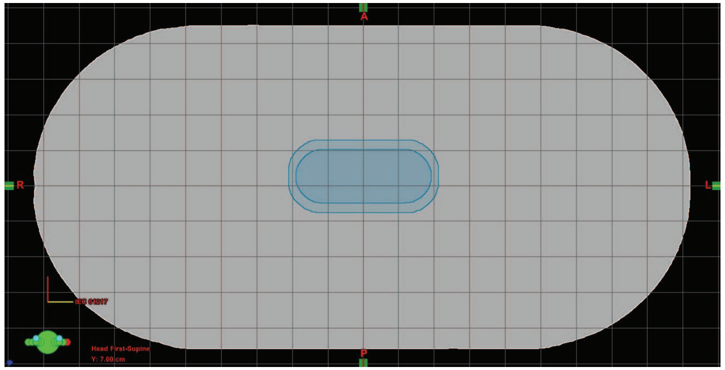
A
R
L
P
IEC 61217
Head First-Supine
Y: 7.00 cm

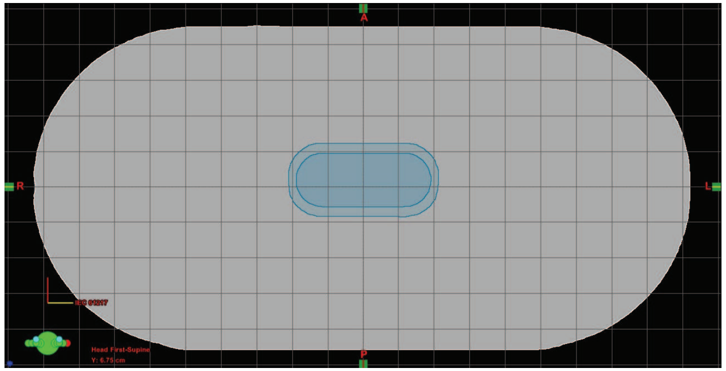
A
R
L
P
IEC 61217
Head First-Supine
Y: 6.75 cm

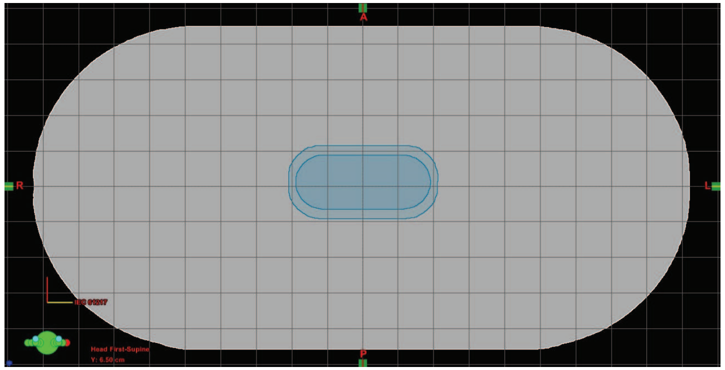
A
R
L
P
IEC 61217
Head First-Supine
Y: 6.50 cm

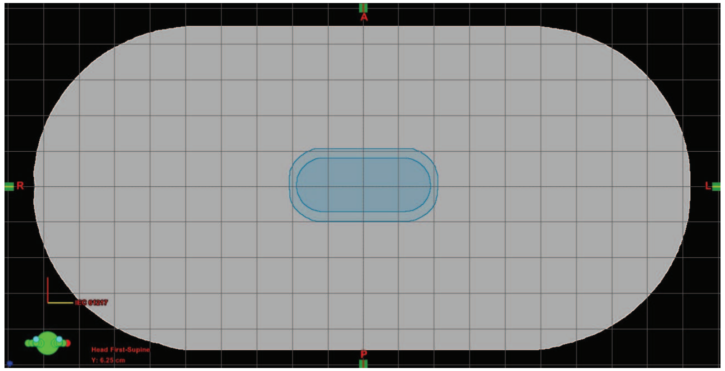
A
R
L
P
IEC 61217
Head First-Supine
Y: 6.25 cm

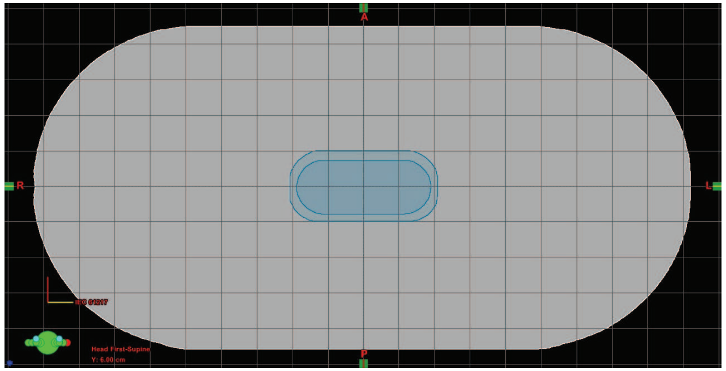
A
R
L
P
IEC 61217
Head First-Supine
Y: 6.00 cm

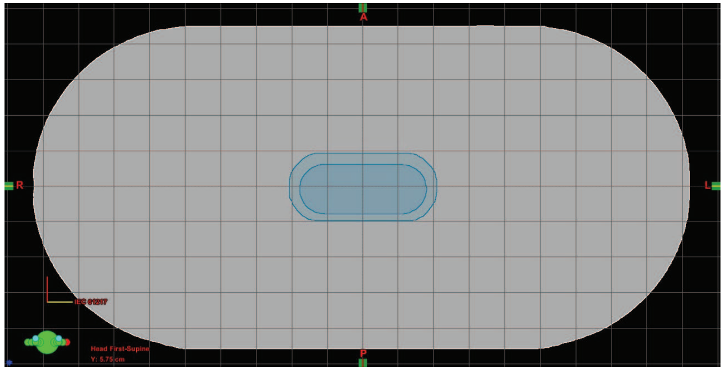
A
R
L
P
IEC 61217
Head First-Supine
Y: 5.75 cm

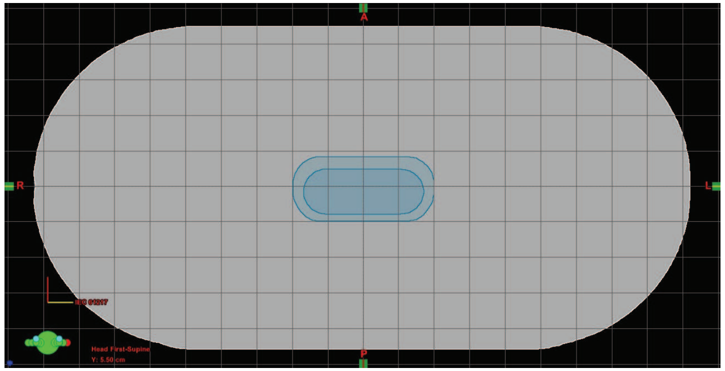
A
R
L
P
IEC 61217
Head First-Supine
Y: 5.50 cm

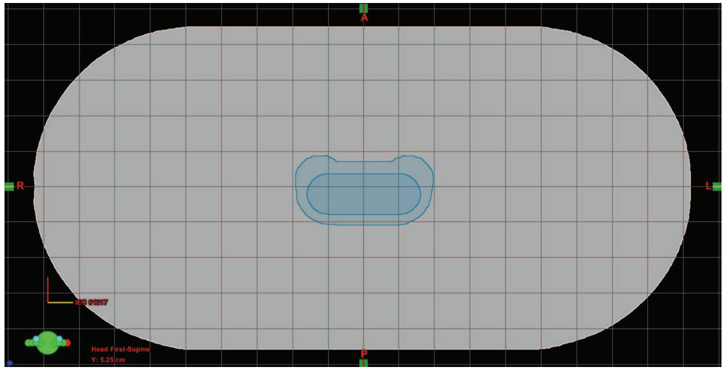
A
R
L
P
IEC 61217
Head First-Supine
Y: 5.25 cm

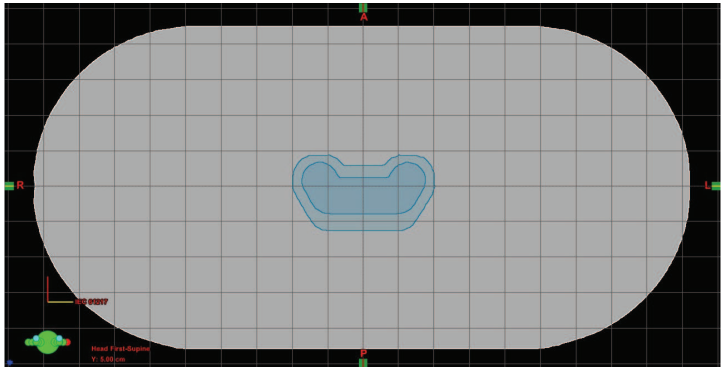
A
R
L
P
IEC 61217
Head First-Supine
Y: 5.00 cm

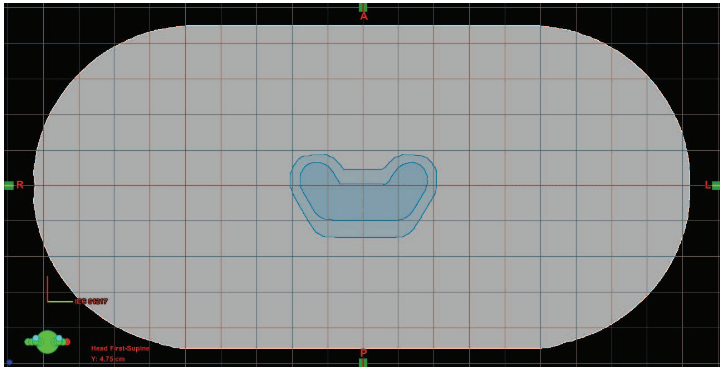
A
R
L
P
IEC 61217
Head First-Supine
Y: 4.75 cm

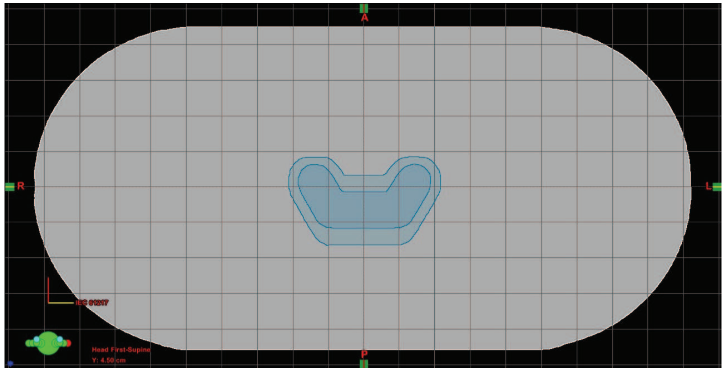
A
R
L
P
IEC 61217
Head First-Supine
Y: 4.50 cm

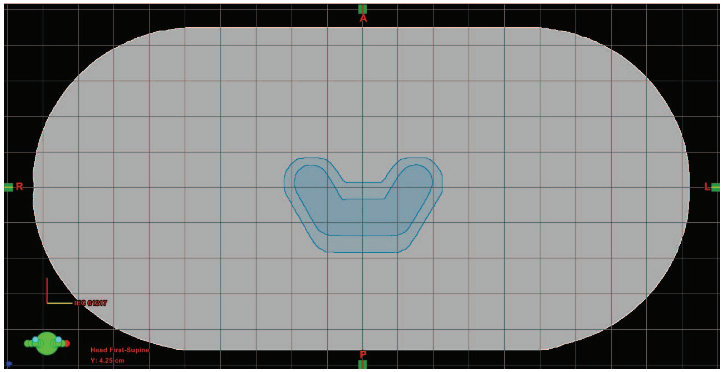
A
R
L
P
IEC 61217
Head First-Supine
Y: 4.25 cm

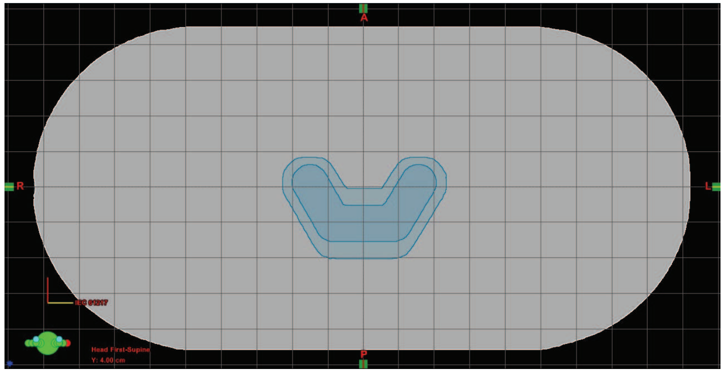
A
R
L
P
IEC 61217
Head First-Supine
Y: 4.00 cm

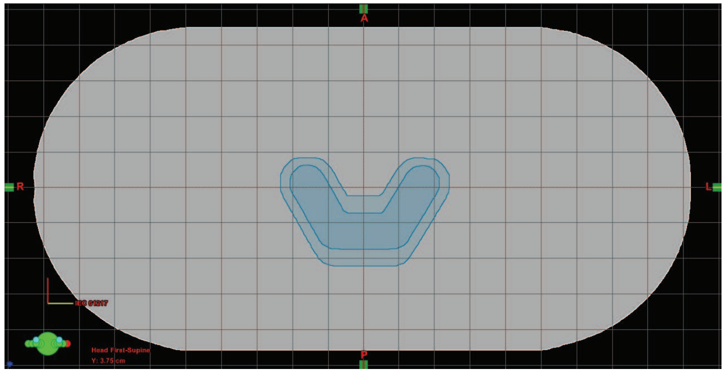
A
R
L
P
IEC 61217
Head First-Supine
Y: 3.75 cm

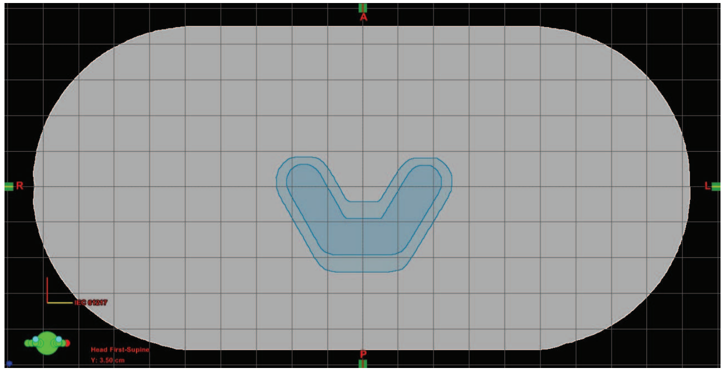
A
R
L
P
IEC 61217
Head First-Supine
Y: 3.50 cm

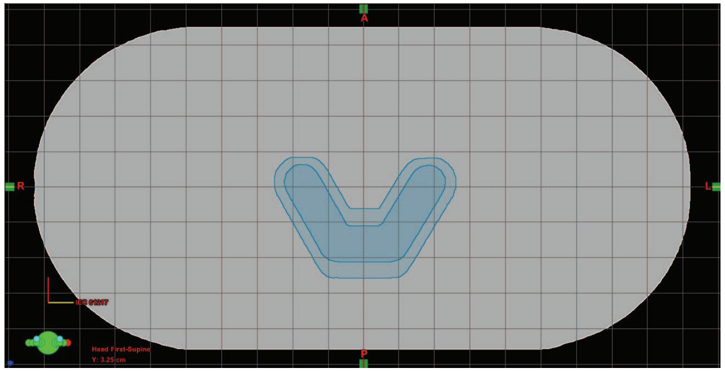
A
R
L
P
IEC 61217
Head First-Supine
Y: 3.25 cm

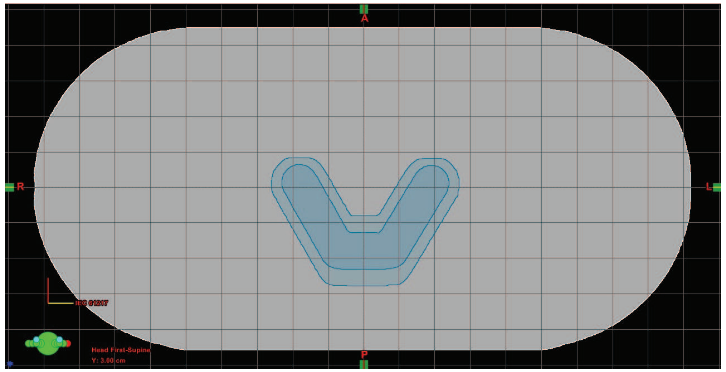
A
R
L
P
IEC 61217
Head First-Supine
Y: 3.00 cm

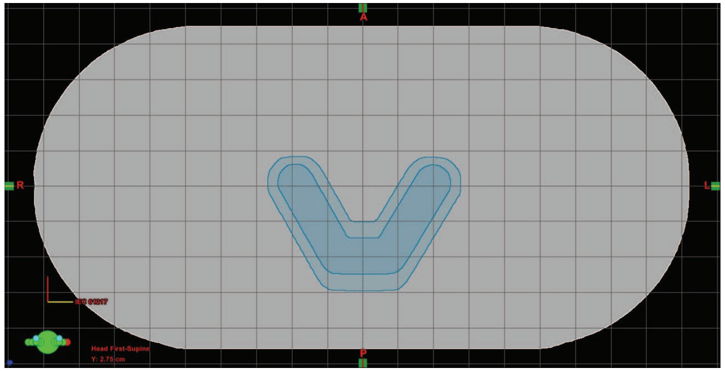
A
R
L
P
IEC 61217
Head First-Supine
Y: 2.75 cm

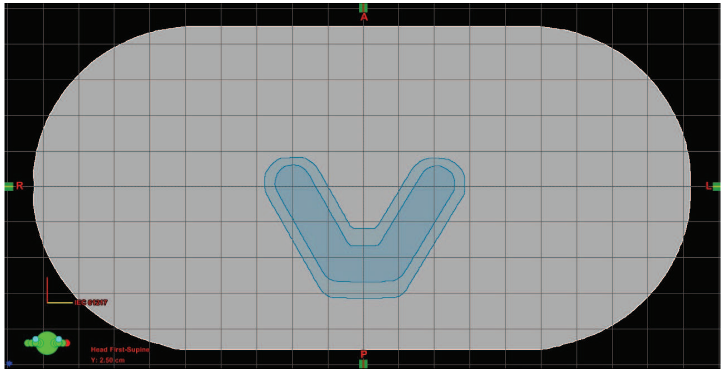
A
R
L
P
IEC 61217
Head First-Supine
Y: 2.50 cm

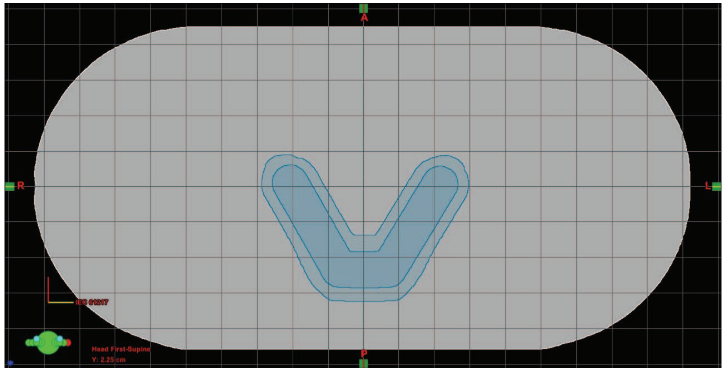
A
R
L
P
IEC 61217
Head First-Supine
Y: 2.25 cm

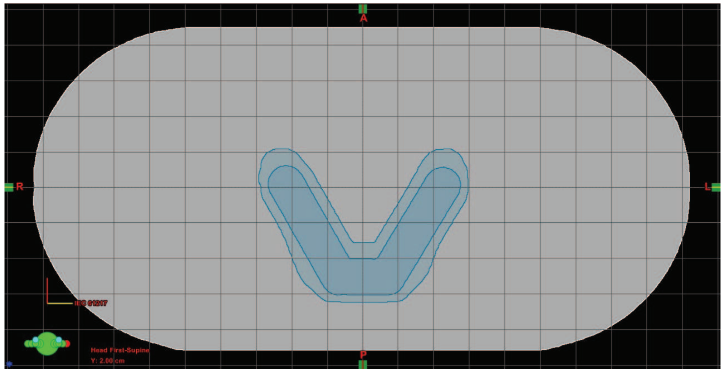
A
R
L
P
IEC 61217
Head First-Supine
Y: 2.00 cm

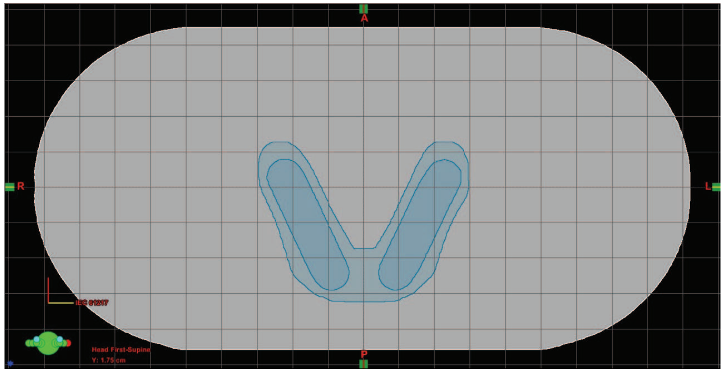
A
R
L
IEC 61217
Head First-Supine
Y: 1.75 cm
P
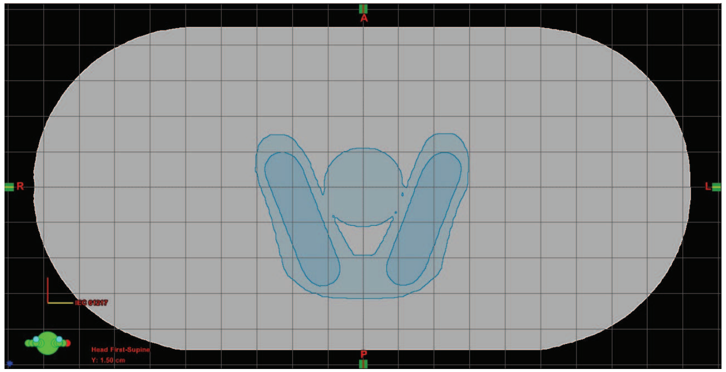
A
R
L
IEC 61217
Head First-Supine
Y: 1.50 cm
P
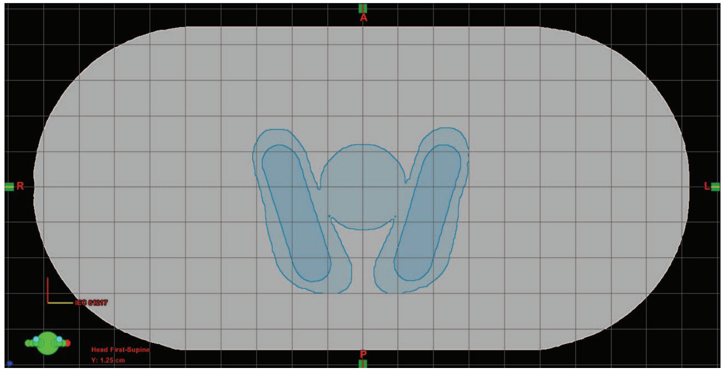
A
R
L
IEC 61217
Head First-Supine
Y: 1.25 cm
P
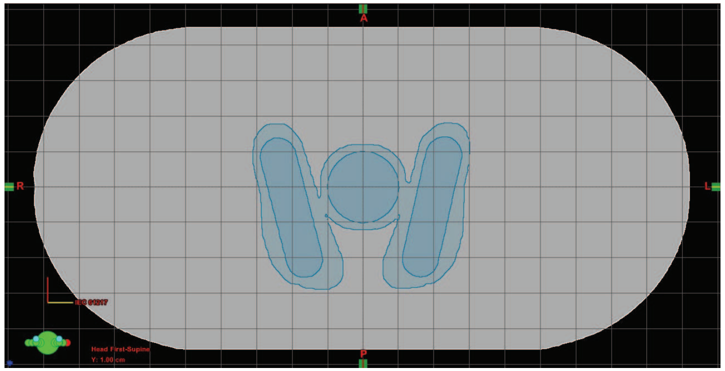
A
R
L
IEC 61217
Head First-Supine
Y: 1.00 cm
P
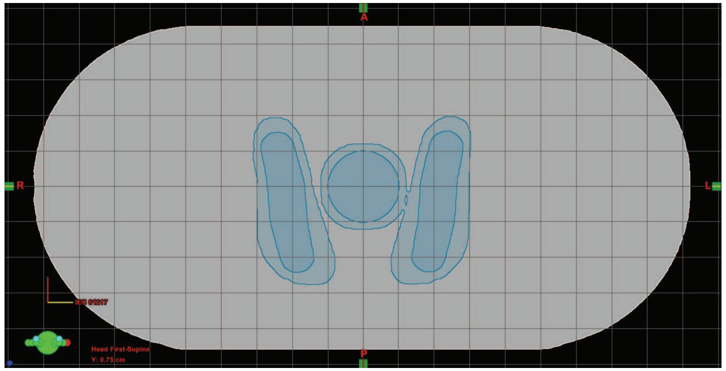
A
R
L
IEC 61217
Head First-Supine
Y: 0.75 cm
P
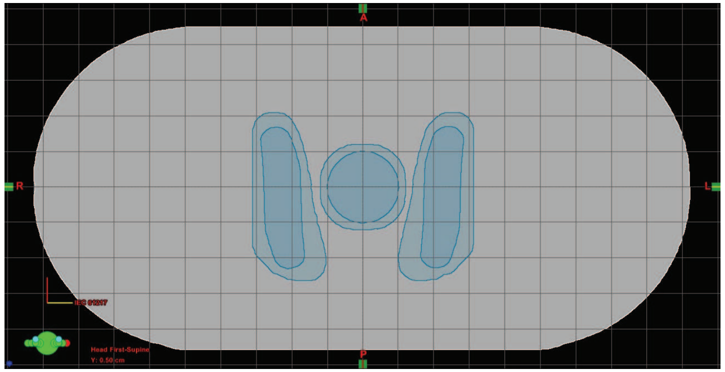
A
R
L
IEC 61217
Head First-Supine
Y: 0.50 cm
P
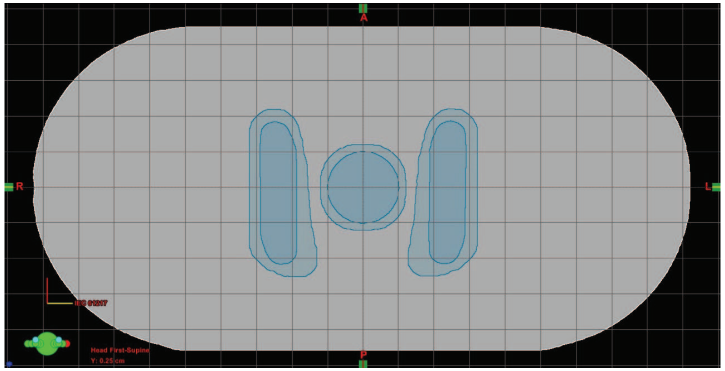
A
R
L
IEC 61217
Head First-Supine
Y: 0.25 cm
P
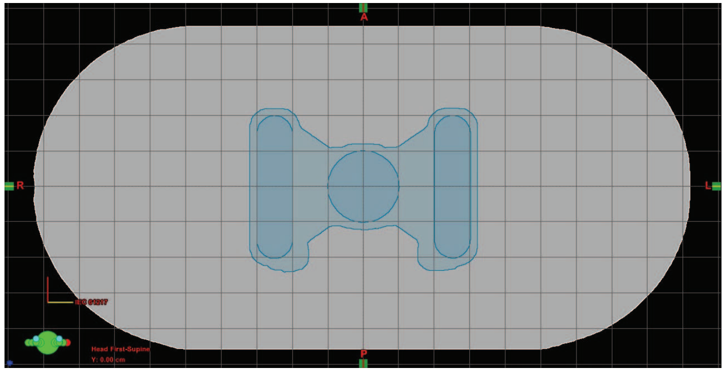
A
R
L
IEC 61217
Head First-Supine
Y: 0.00 cm
P
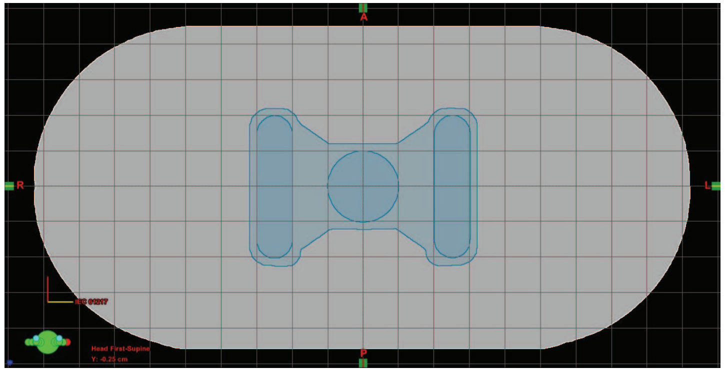
A
R
L
IEC 61217
Head First-Supine
Y: -0.25 cm
P

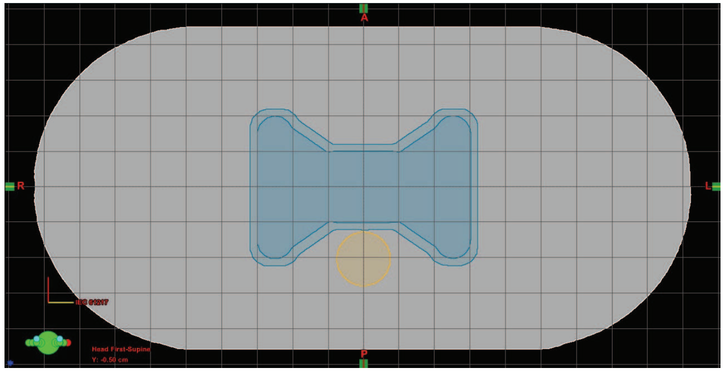
A
R
L
P
IEC 61217
Head First-Supine
Y: -0.50 cm

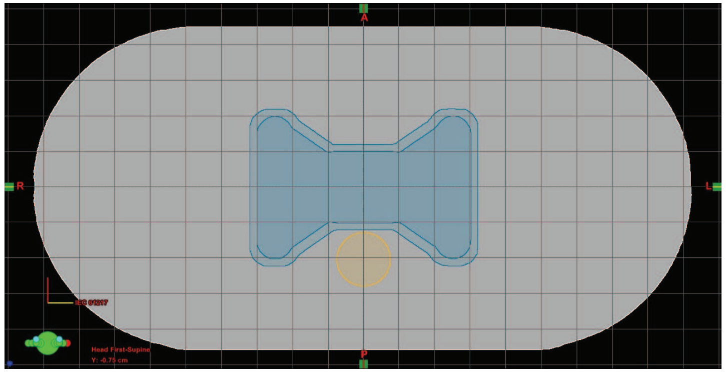
A
R
L
P
IEC 61217
Head First-Supine
Y: -0.75 cm

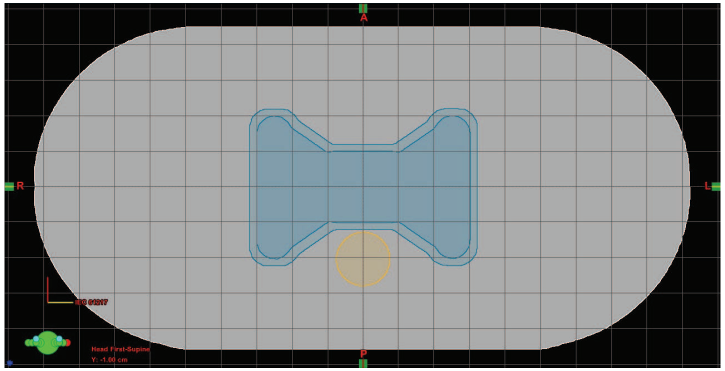
A
R
L
P
IEC 61217
Head First-Supine
Y: -1.00 cm

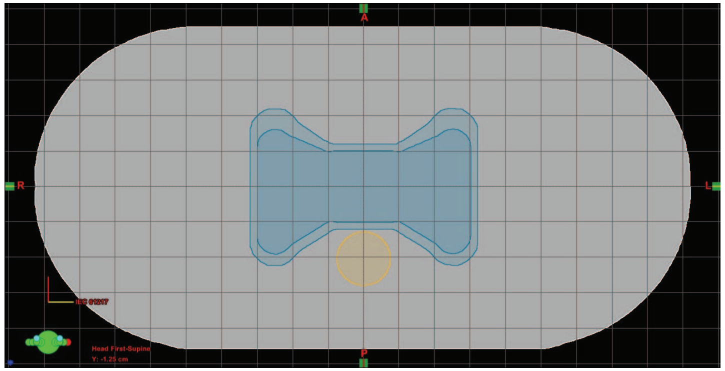
A
R
L
P
IEC 61217
Head First-Supine
Y: -1.25 cm

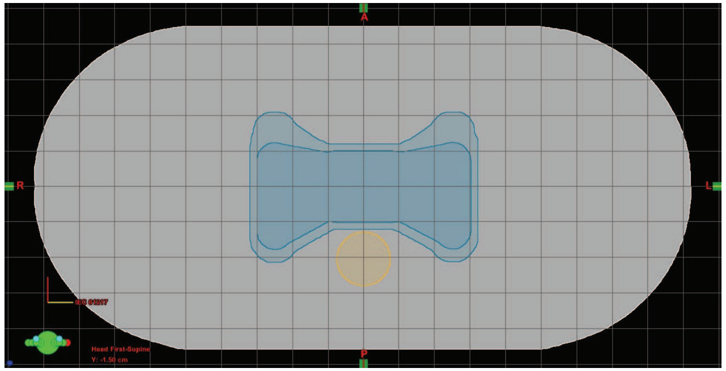
A
R
L
P
IEC 61217
Head First-Supine
Y: -1.50 cm

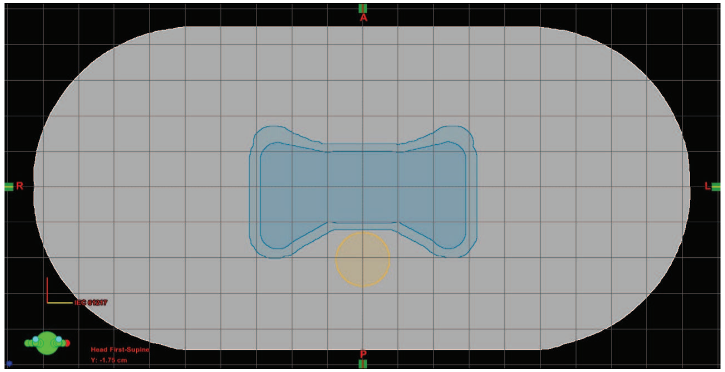
A
R
L
P
IEC 61217
Head First-Supine
Y: -1.75 cm

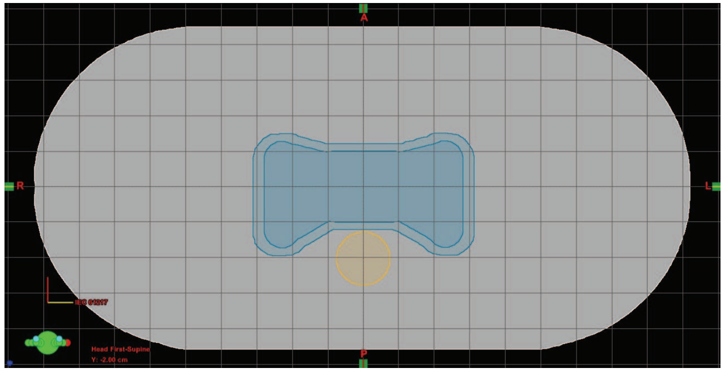
A
R
L
P
IEC 61217
Head First-Supine
Y: -2.00 cm

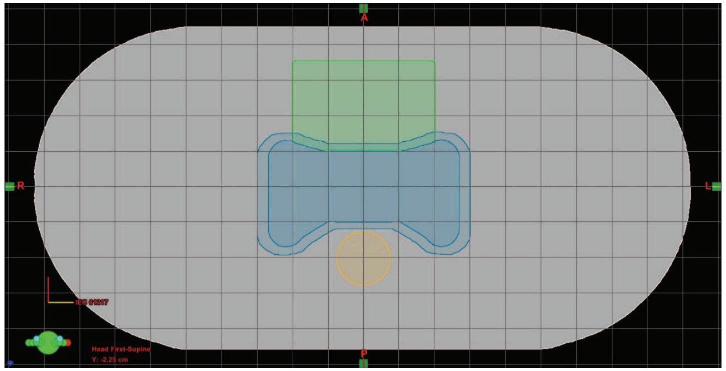
A
R
L
P
IEC 61217
Head First-Supine
Y: -2.25 cm

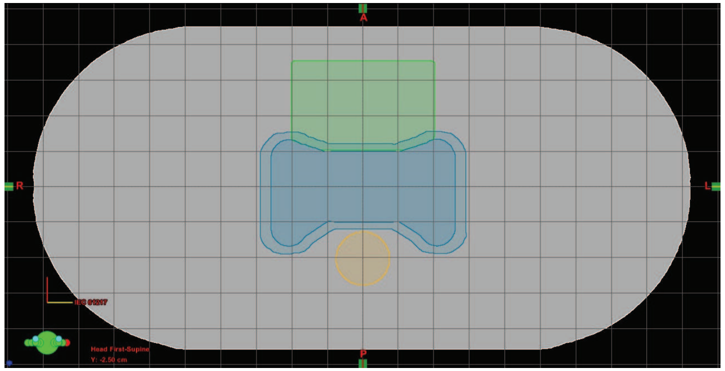
A
R
L
P
IEC 61217
Head First-Supine
Y: -2.50 cm

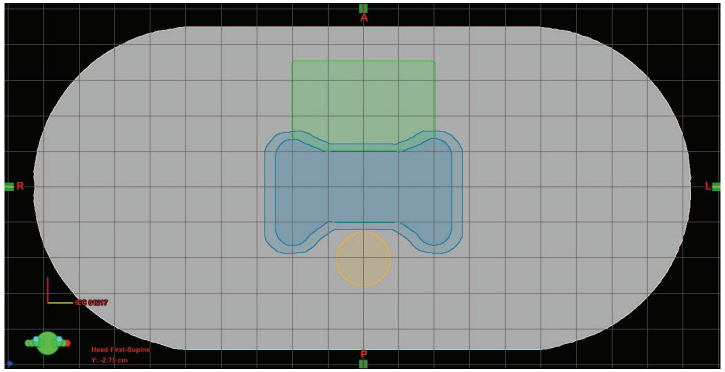
A
R
L
P
IEC 61217
Head First-Supine
Y: -2.75 cm

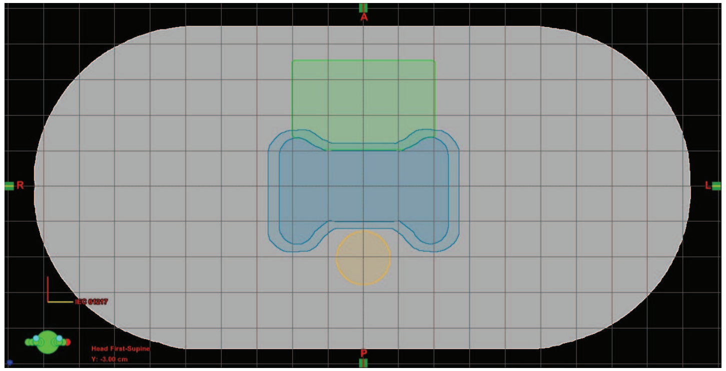
A
R
L
P
IEC 61217
Head First-Supine
Y: -3.00 cm

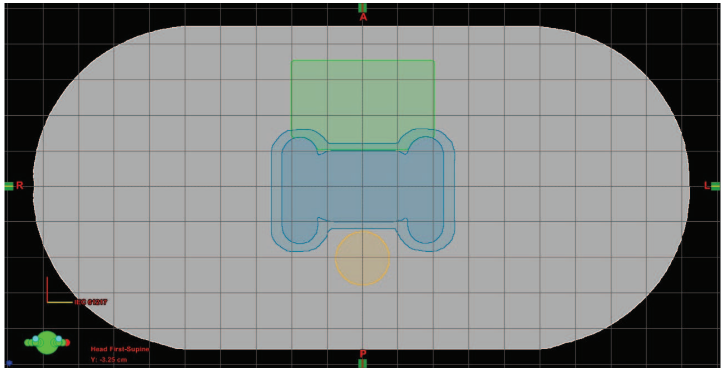
A
R
L
P
IEC 61217
Head First-Supine
Y: -3.25 cm

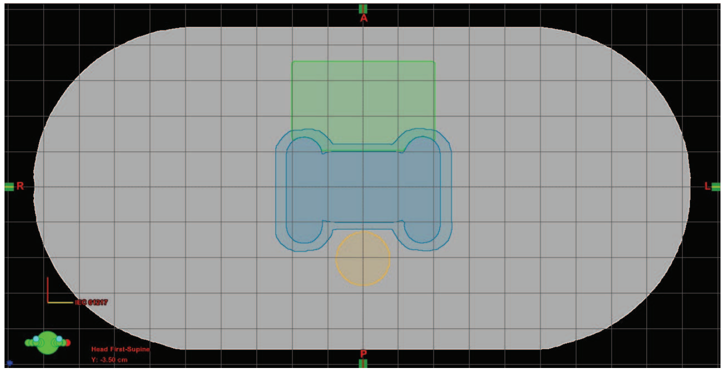
A
R
L
P
IEC 61217
Head First-Supine
Y: -3.50 cm

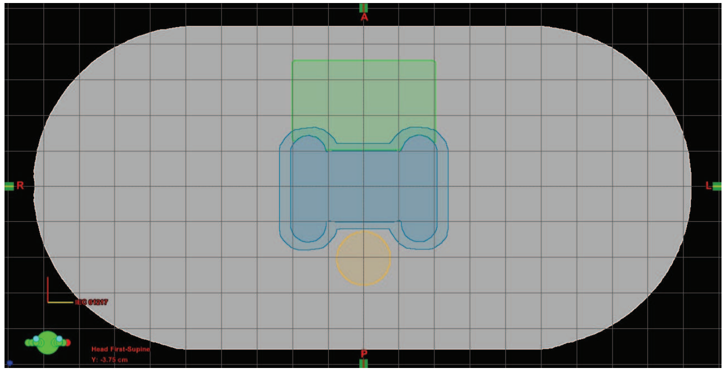
A
R
L
P
IEC 61217
Head First-Supine
Y: -3.75 cm

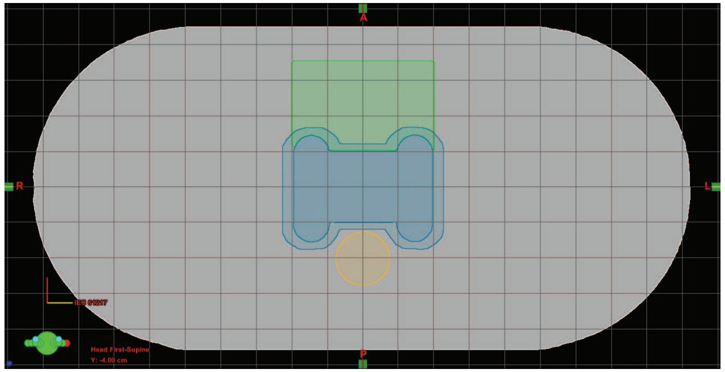
A
R
L
P
IEC 61217
Head First-Supine
Y: -4.00 cm

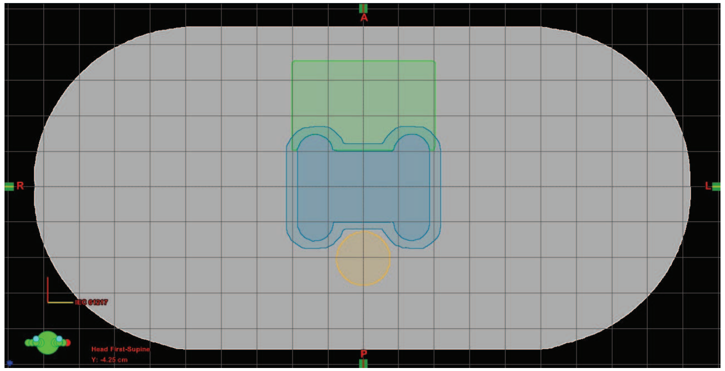
A
R
L
P
IEC 61217
Head First-Supine
Y: -4.25 cm

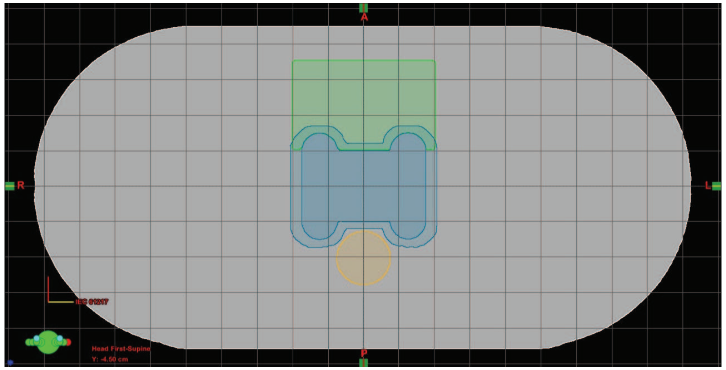
A
R
L
P
IEC 61217
Head First-Supine
Y: -4.50 cm

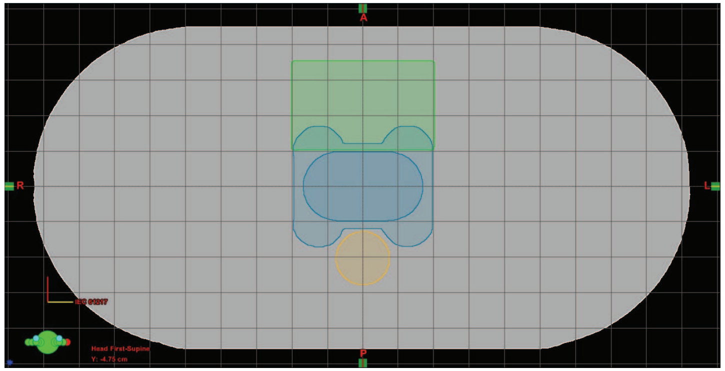
A
R
L
P
IEC 61217
Head First-Supine
Y: -4.75 cm

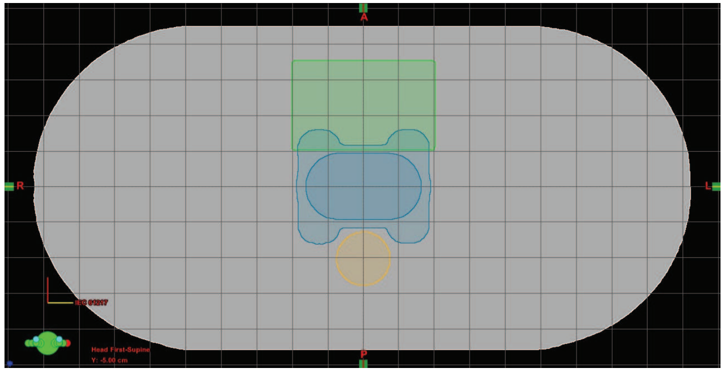
A
R
L
P
IEC 61217
Head First-Supine
Y: -5.00 cm

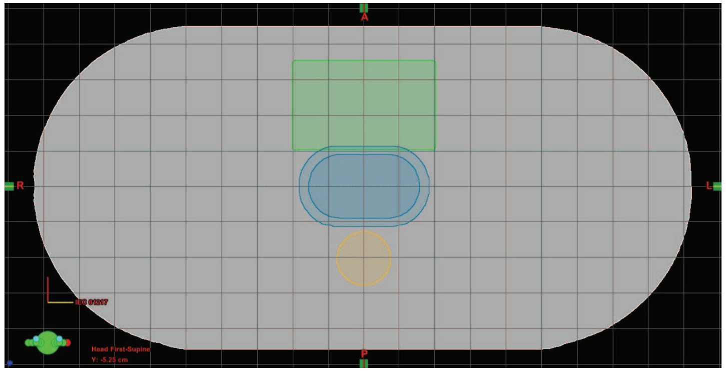
A
R
L
P
IEC 61217
Head First-Supine
Y: -5.25 cm

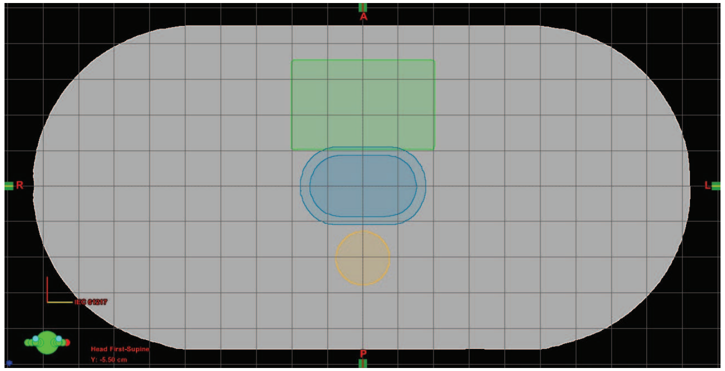
A
R
L
P
IEC 61217
Head First-Supine
Y: -5.50 cm

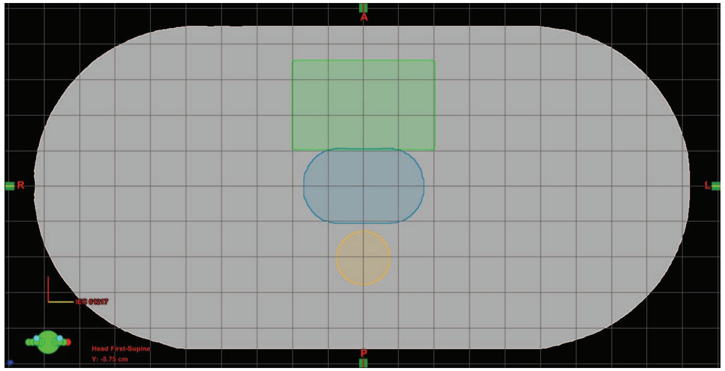
A
R
L
P
IEC 61217
Head First-Supine
Y: -5.75 cm

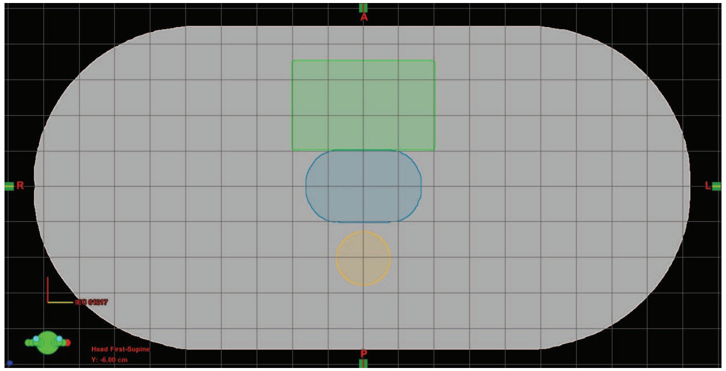
A
R
L
P
IEC 61217
Head First-Supine
Y: -6.00 cm
