# Supplementary material for: Dosimetric comparison of three-dimensional conformal radiotherapy versus volumetric-arc radiotherapy in cervical cancer treatment: applying the central-shielding principle to modern technology
Source: J Radiat Res. 2018 Jul 21;59(5):639–48. doi: 10.1093/jrr/rry054 (PMC6151642; doi:10.1093/jrr/rry054)
Supplement: Supplementary Data [file rry054_supplementary_figure_2_small.pdf]

Supplementary Figure 2: Axial images of the $\text{CTV-CS}_{\text{phantom}}$ (red), $\text{bladder}_{\text{phantom}}$ (green), $\text{rectum}_{\text{phantom}}$ (orange), and $\text{PTV-CS}_{\text{phantom}}$ (light red) on a dosimetric phantom. The images are shown with grids of 2 cm and in an interval of 2.5 mm in crainal-caudal direction. The 4-cm width at the central part of the $\text{CTV-WP}_{\text{phantom}}$ (uterus corpus, upper vagina, and some parametrial tissue of the phantom) was removed to create $\text{CTV-CS}_{\text{phantom.}}$

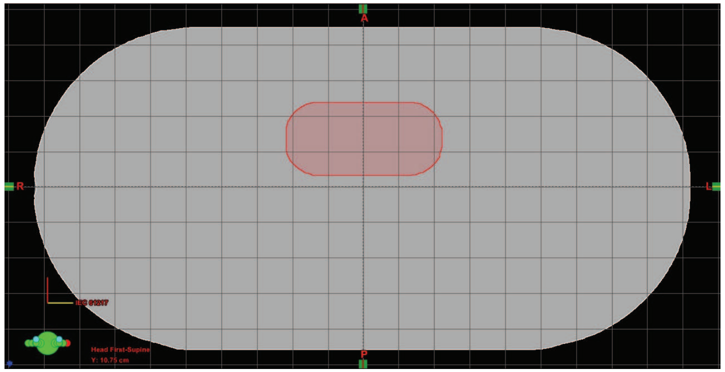
A
R
L
P
IEC 61217
Head First-Supine
Y: 10.75 cm
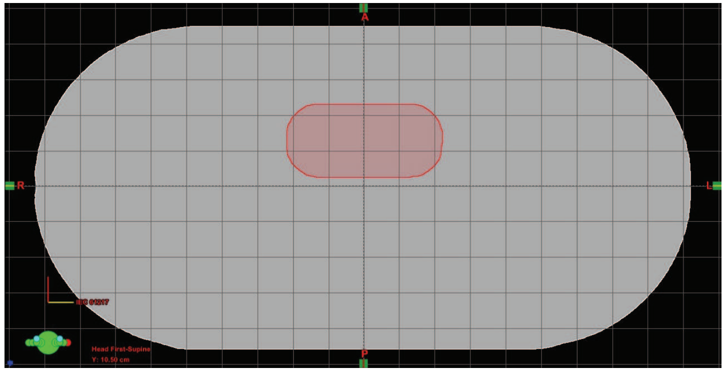
A
R
L
P
IEC 61217
Head First-Supine
Y: 10.50 cm
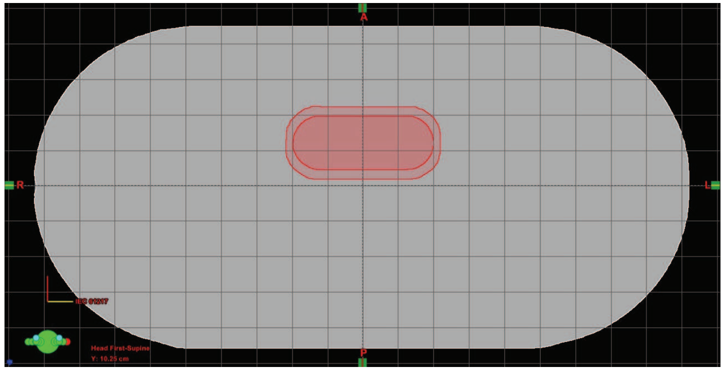
A
R
L
P
IEC 61217
Head First-Supine
Y: 10.25 cm
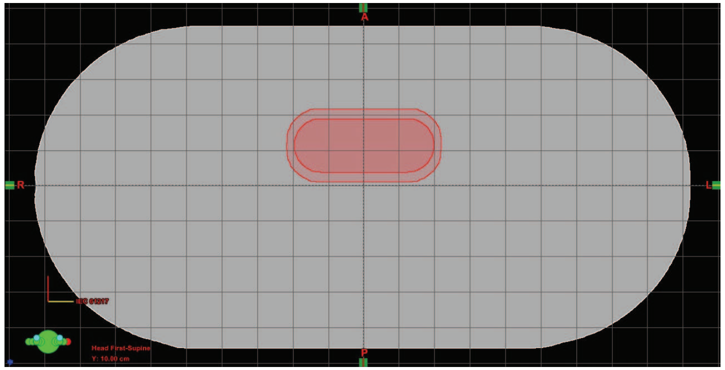
A
R
L
P
IEC 61217
Head First-Supine
Y: 10.00 cm
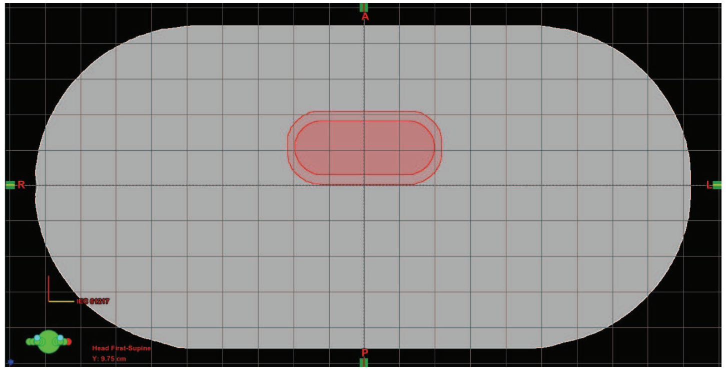
A
R
L
P
IEC 61217
Head First-Supine
Y: 9.75 cm
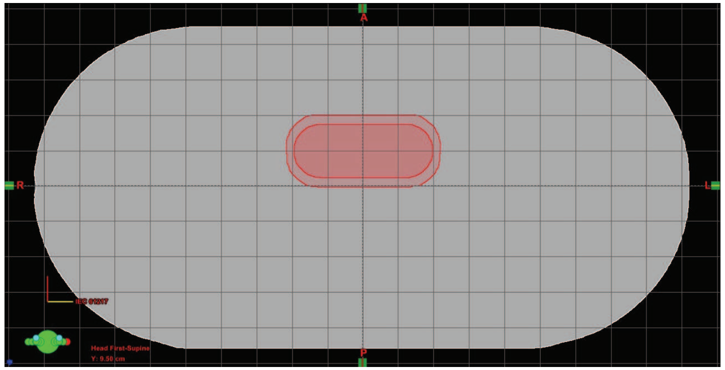
A
R
L
P
IEC 61217
Head First-Supine
Y: 9.50 cm
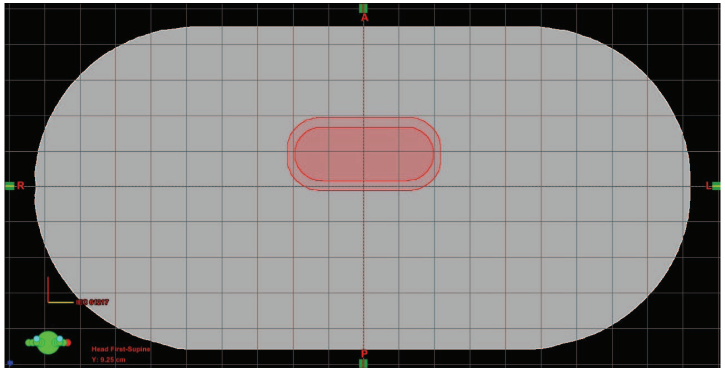
A
R
L
P
IEC 61217
Head First-Supine
Y: 9.25 cm
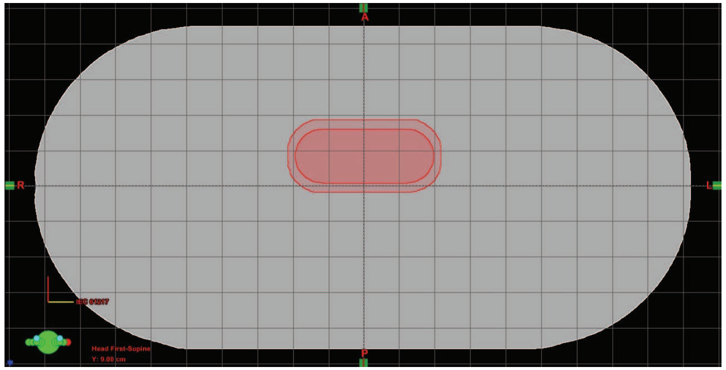
A
R
L
P
IEC 61217
Head First-Supine
Y: 9.00 cm
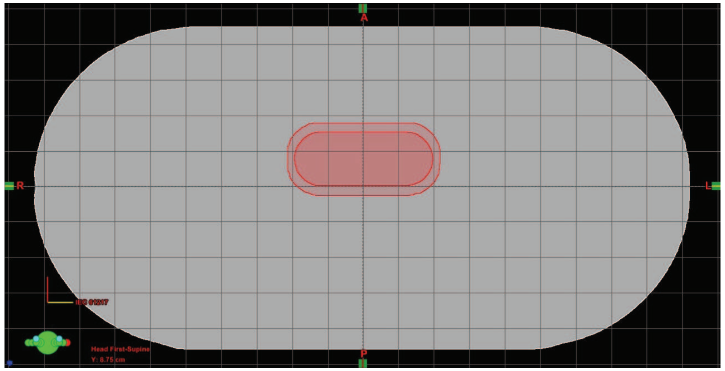
A
R
L
P
IEC 61217
Head First-Supine
Y: 8.75 cm

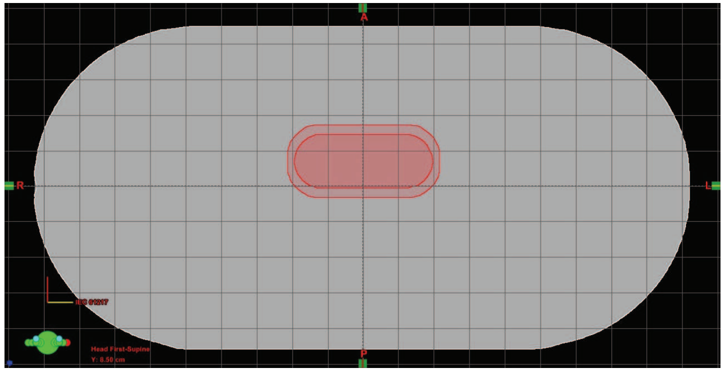
A
R
L
P
IEC 61217
Head First-Supine
Y: 8.50 cm

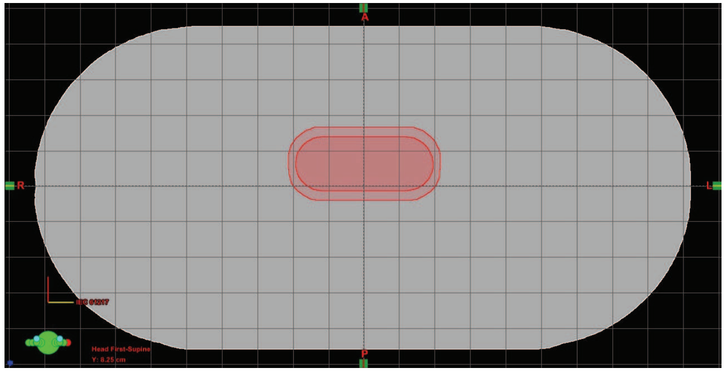
A
R
L
P
IEC 61217
Head First-Supine
Y: 8.25 cm

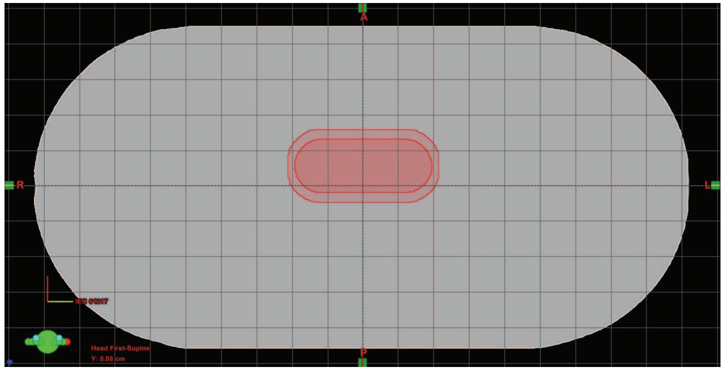
A
R
L
P
IEC 61217
Head First-Supine
Y: 8.00 cm

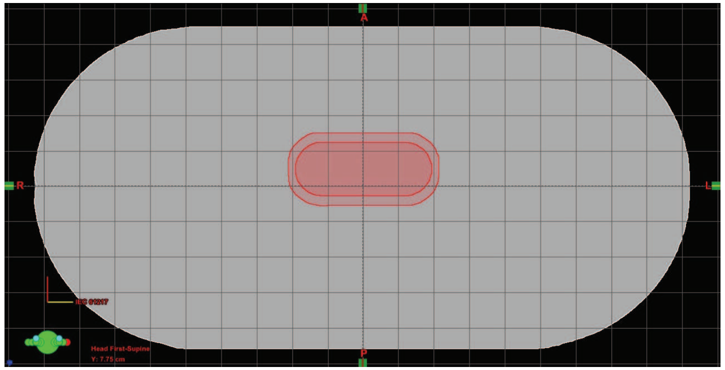
A
R
L
P
IEC 61217
Head First-Supine
Y: 7.75 cm

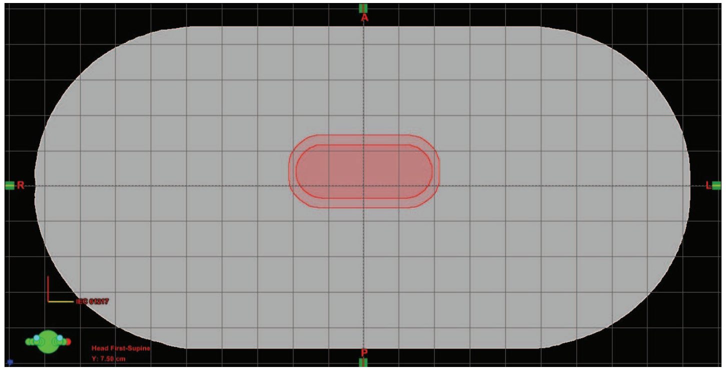
A
R
L
P
IEC 61217
Head First-Supine
Y: 7.50 cm

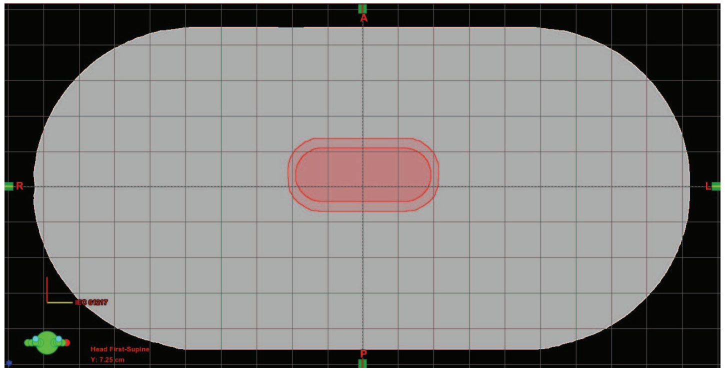
A
R
L
P
IEC 61217
Head First-Supine
Y: 7.25 cm

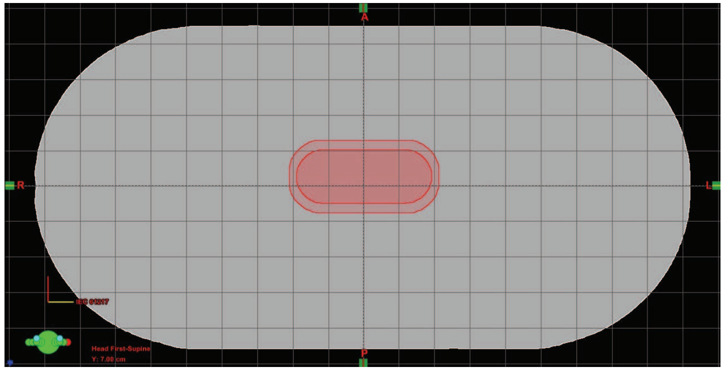
A
R
L
P
IEC 61217
Head First-Supine
Y: 7.00 cm

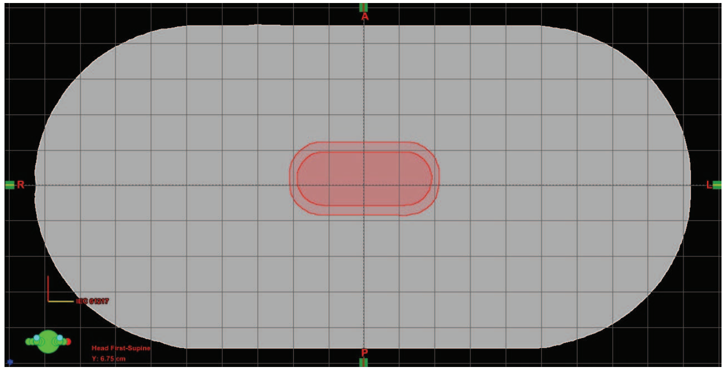
A
R
L
P
IEC 61217
Head First-Supine
Y: 6.75 cm

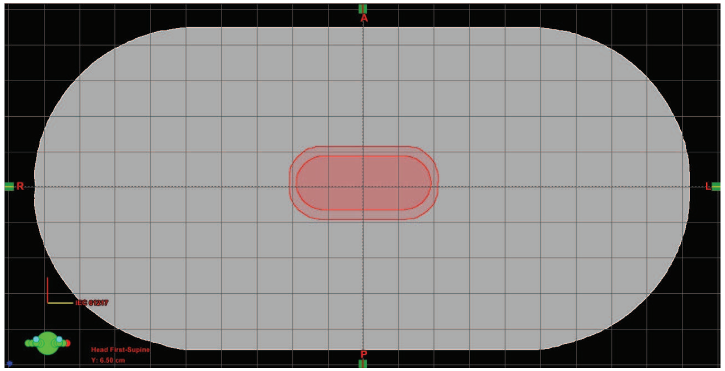
A
R
L
P
IEC 61217
Head First-Supine
Y: 6.50 cm

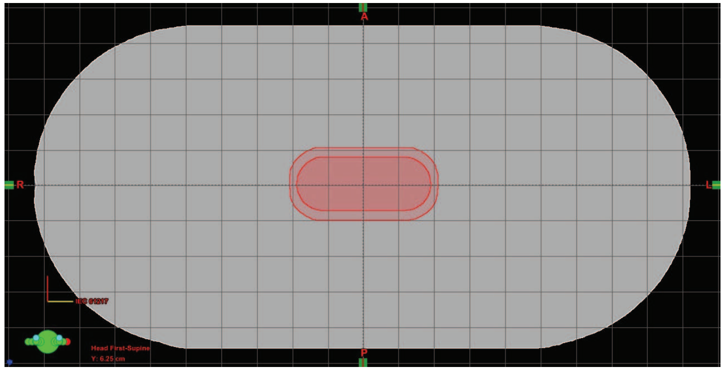
A
R
L
P
IEC 61217
Head First-Supine
Y: 6.25 cm

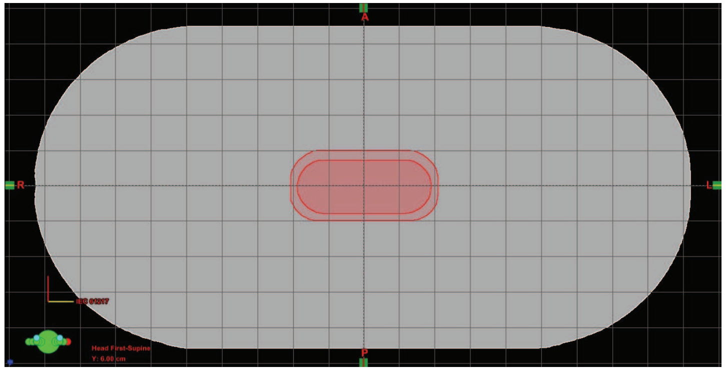
A
R
L
P
IEC 61217
Head First-Supine
Y: 6.00 cm

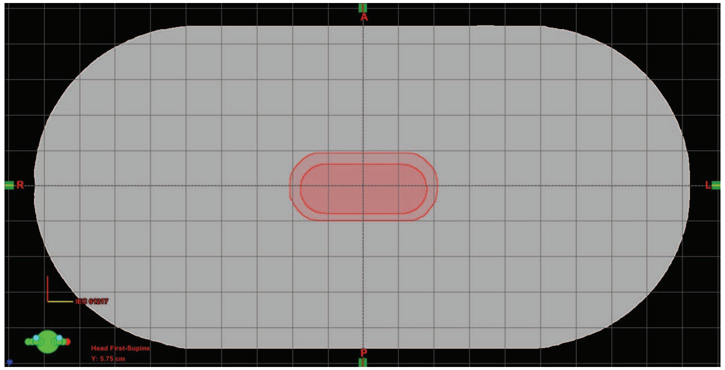
A
R
L
P
IEC 61217
Head First-Supine
Y: 5.75 cm

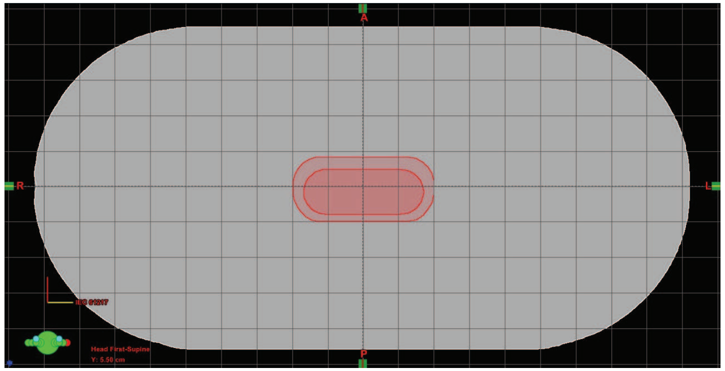
A
R
L
P
IEC 61217
Head First-Supine
Y: 5.50 cm

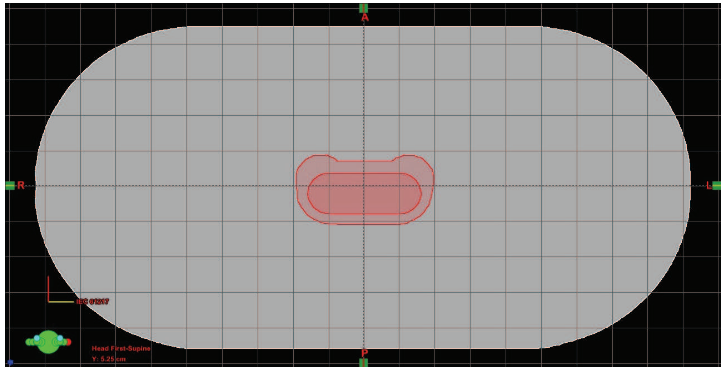
A
R
L
P
IEC 61217
Head First-Supine
Y: 5.25 cm

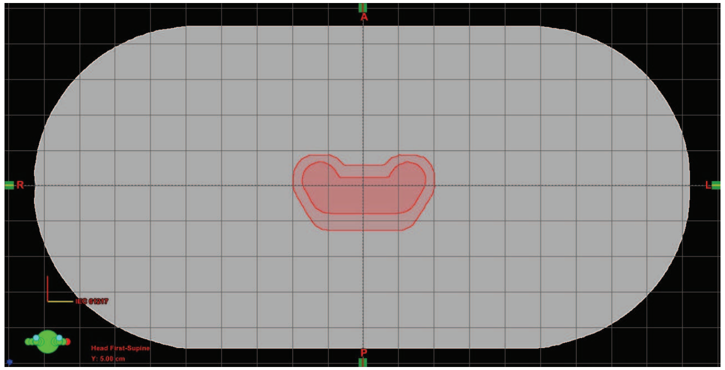
A
R
L
P
IEC 61217
Head First-Supine
Y: 5.00 cm

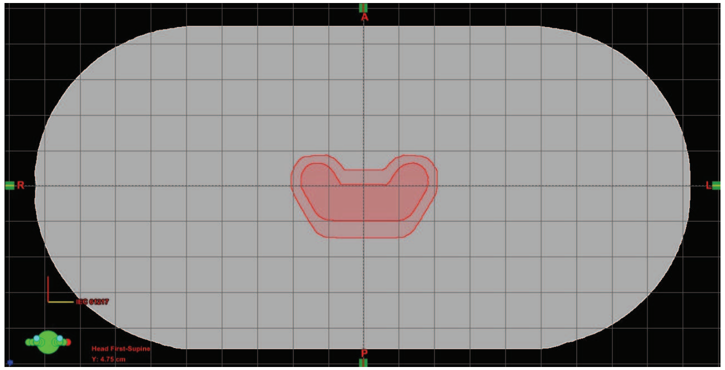
A
R
L
P
IEC 61217
Head First-Supine
Y: 4.75 cm

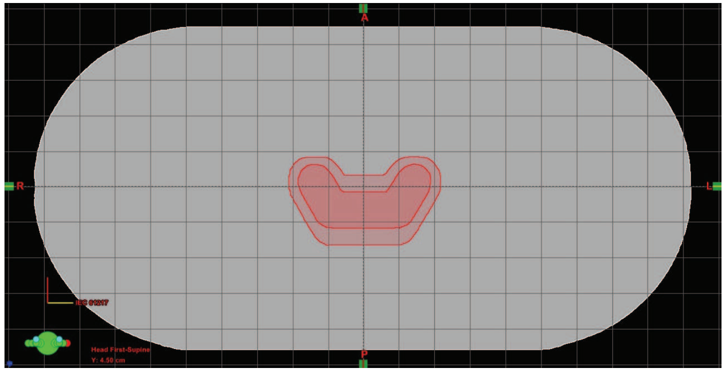
A
R
L
P
IEC 61217
Head First-Supine
Y: 4.50 cm

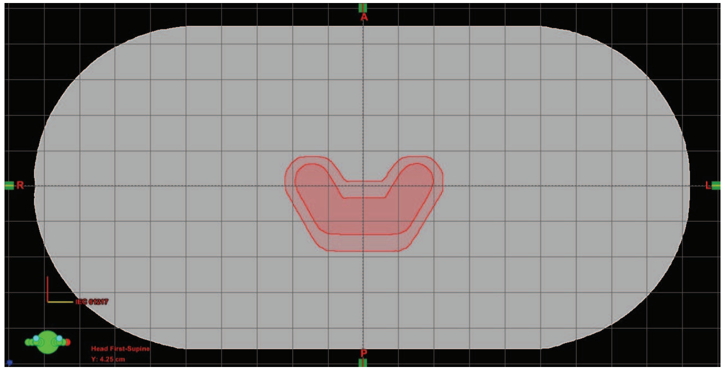
A
R
L
P
IEC 61217
Head First-Supine
Y: 4.25 cm

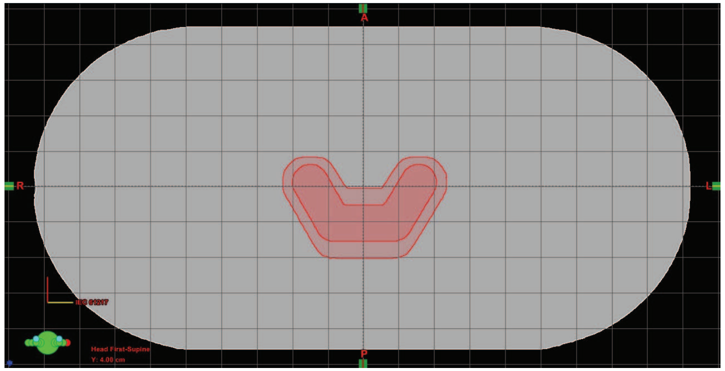
A
R
L
P
IEC 61217
Head First-Supine
Y: 4.00 cm

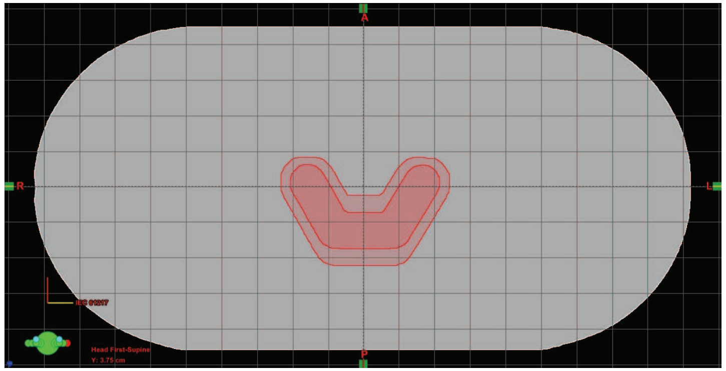
A
R
L
P
IEC 61217
Head First-Supine
Y: 3.75 cm

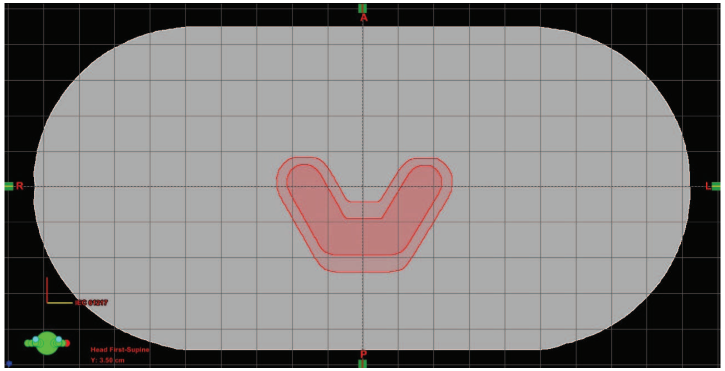
A
R
L
P
IEC 61217
Head First-Supine
Y: 3.50 cm

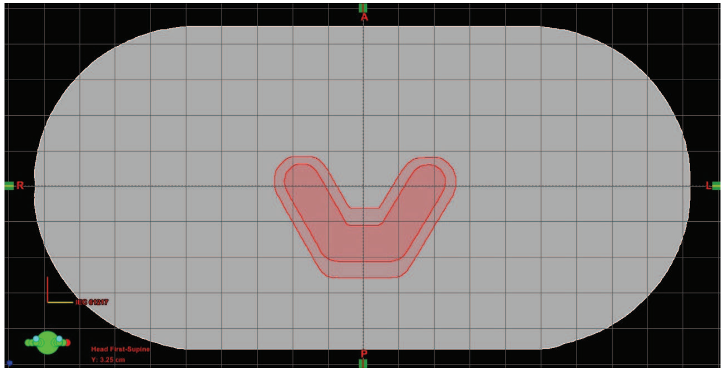
A
R
L
P
IEC 61217
Head First-Supine
Y: 3.25 cm

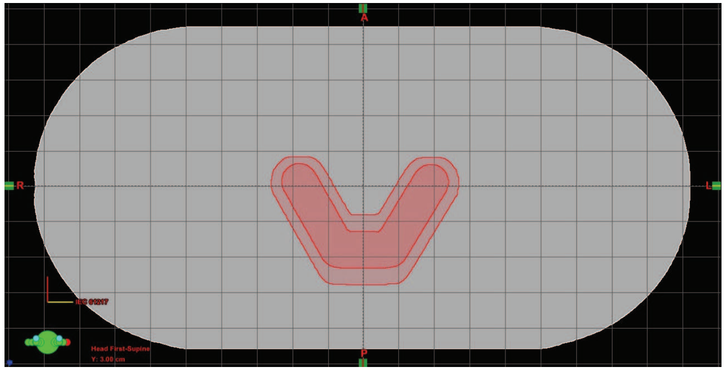
A
R
L
P
IEC 61217
Head First-Supine
Y: 3.00 cm

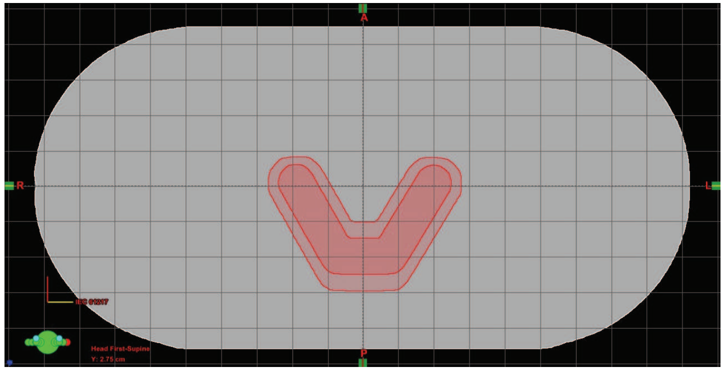
A
R
L
P
IEC 61217
Head First-Supine
Y: 2.75 cm

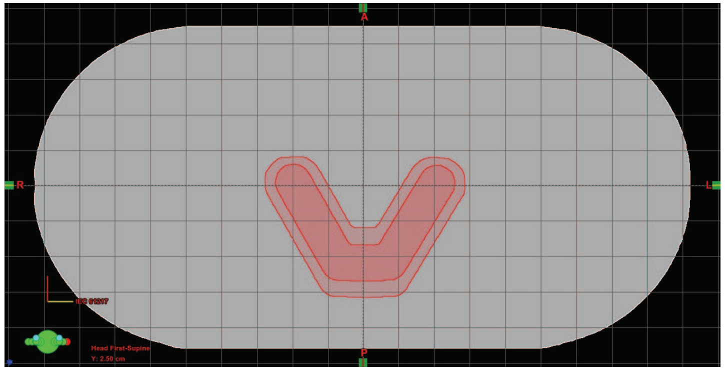
A
R
L
P
IEC 61217
Head First-Supine
Y: 2.50 cm

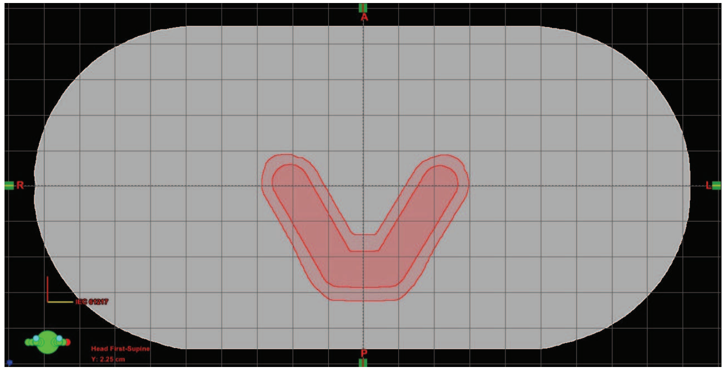
A
R
L
P
IEC 61217
Head First-Supine
Y: 2.25 cm

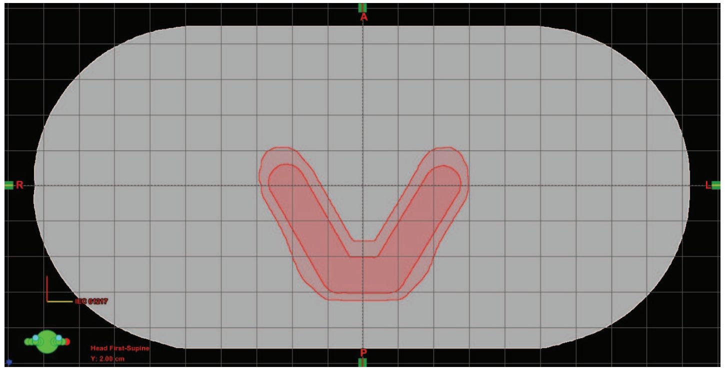
A
R
L
P
IEC 61217
Head First-Supine
Y: 2.00 cm

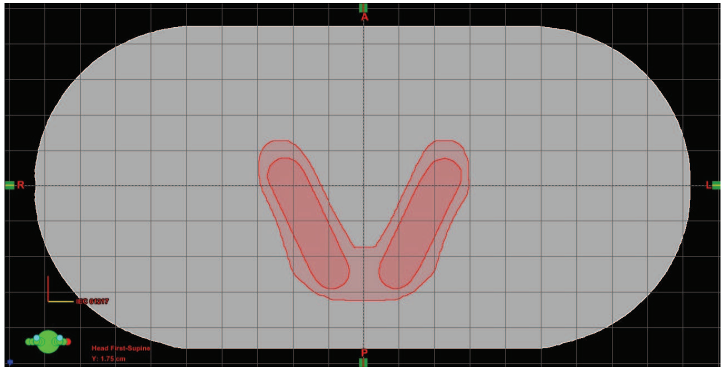
A
R
L
P
IEC 61217
Head First-Supine
Y: 1.75 cm

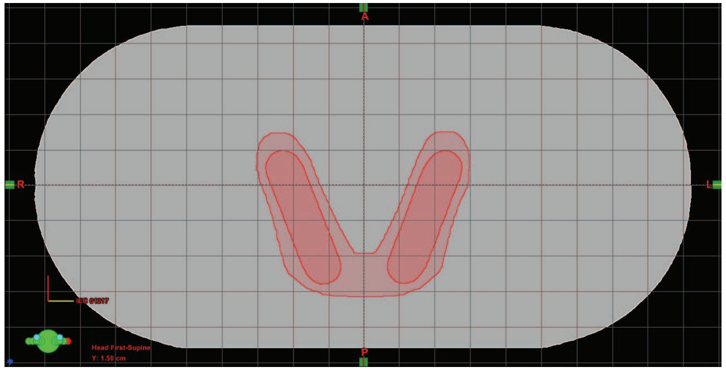
A
R
L
P
IEC 61217
Head First-Supine
Y: 1.50 cm

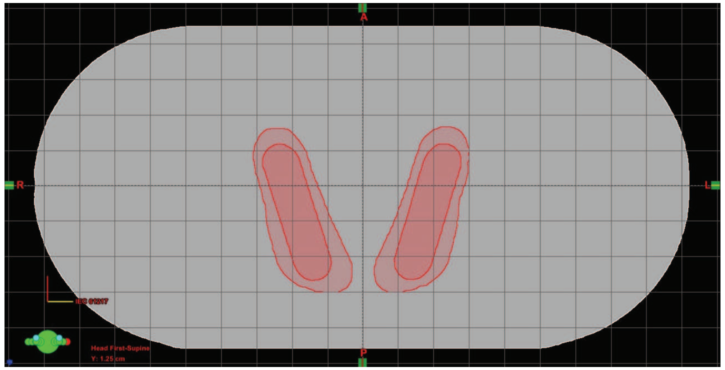
A
R
L
P
IEC 61217
Head First-Supine
Y: 1.25 cm

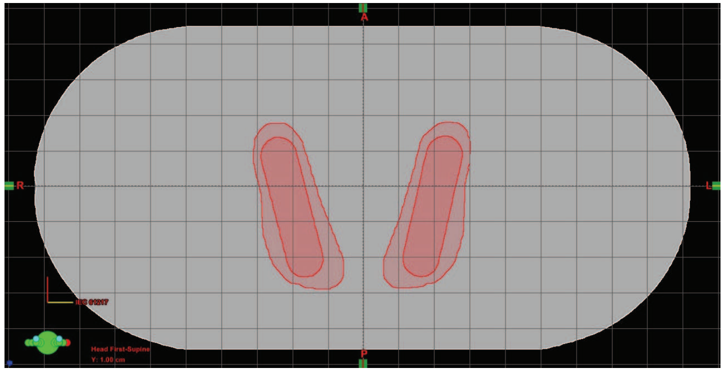
A
R
L
P
IEC 61217
Head First-Supine
Y: 1.00 cm

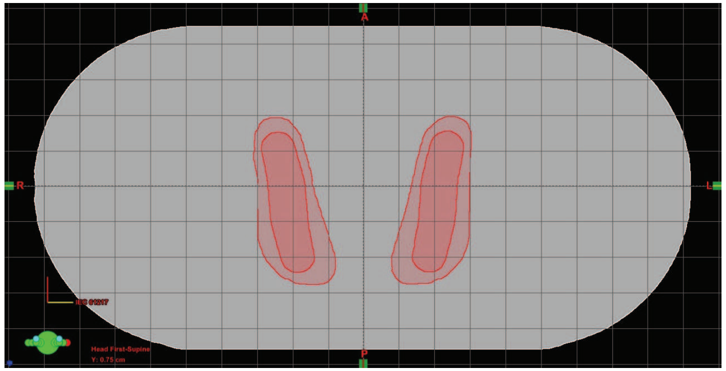
A
R
L
P
IEC 61217
Head First-Supine
Y: 0.75 cm

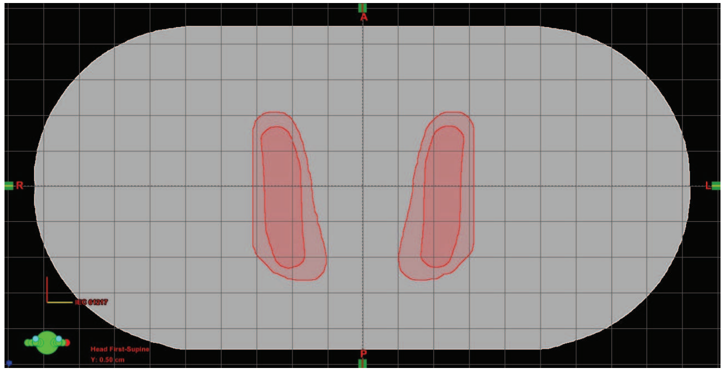
A
R
L
P
IEC 61217
Head First-Supine
Y: 0.50 cm

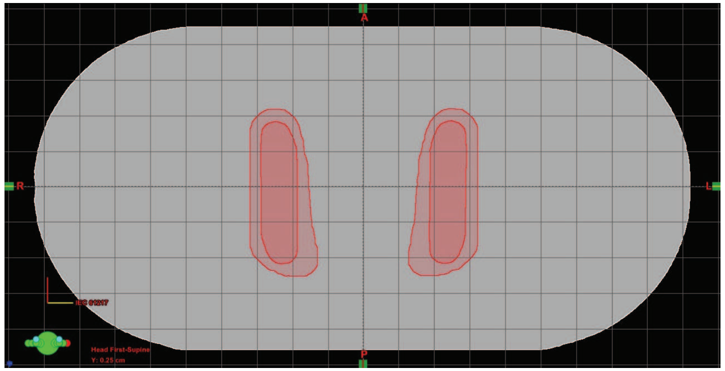
A
R
L
P
IEC 61217
Head First-Supine
Y: 0.25 cm

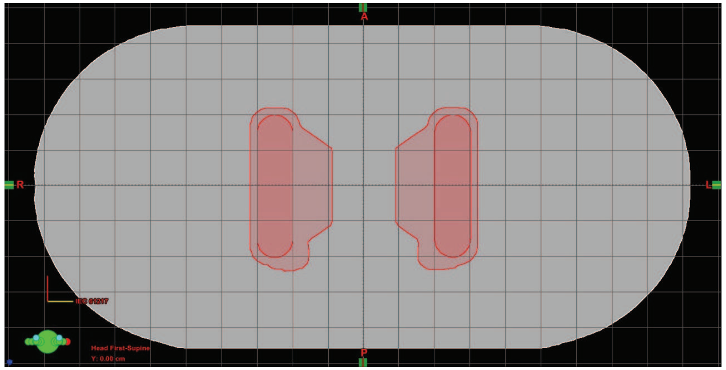
A
R
L
P
IEC 61217
Head First-Supine
Y: 0.00 cm

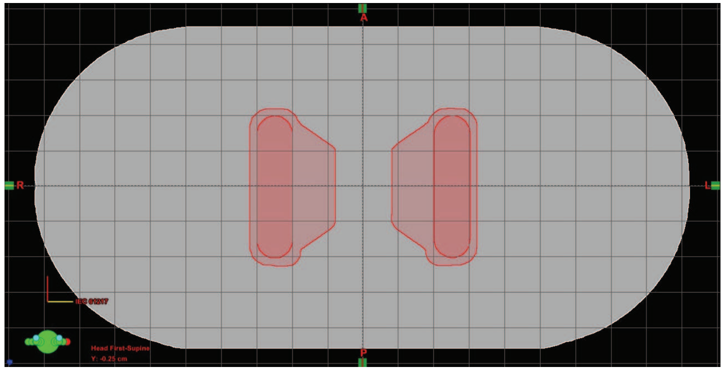
A
R
L
P
IEC 61217
Head First-Supine
Y: -0.25 cm

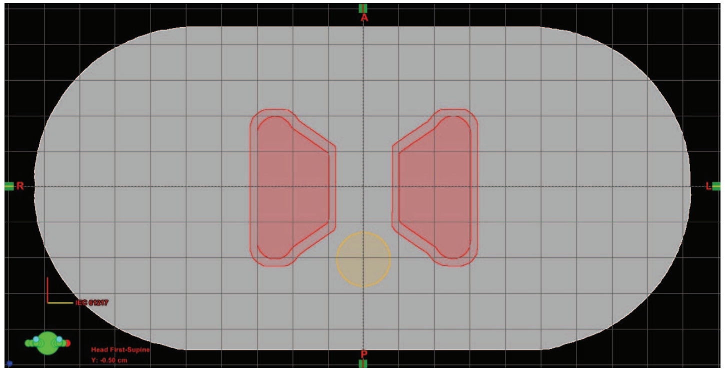
A
R
L
P
IEC 61217
Head First-Supine
Y: -0.50 cm

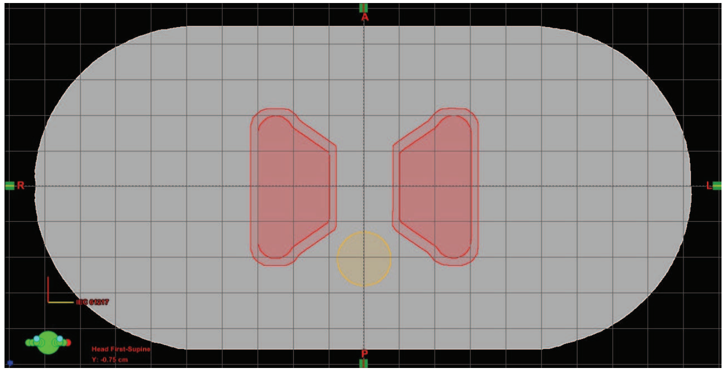
A
R
L
P
IEC 61217
Head First-Supine
Y: -0.75 cm

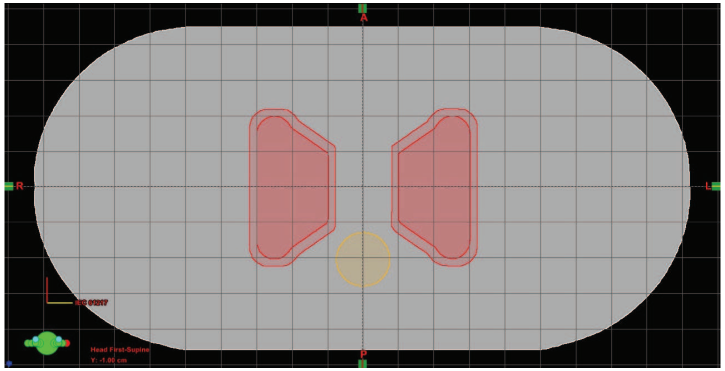
A
R
L
P
IEC 61217
Head First-Supine
Y: -1.00 cm

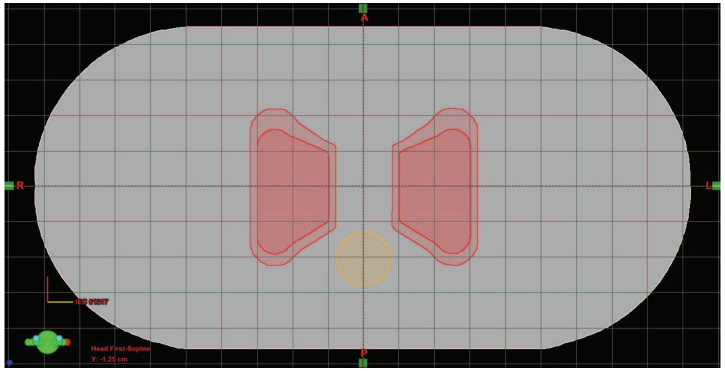
A
R
L
P
IEC 61217
Head First-Supine
Y: -1.25 cm

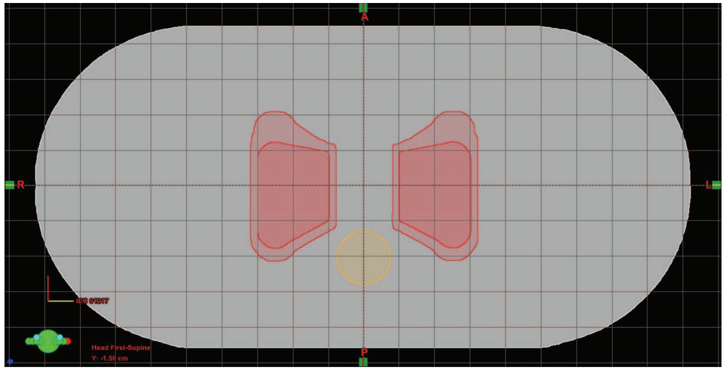
A
R
L
P
IEC 61217
Head First-Supine
Y: -1.50 cm

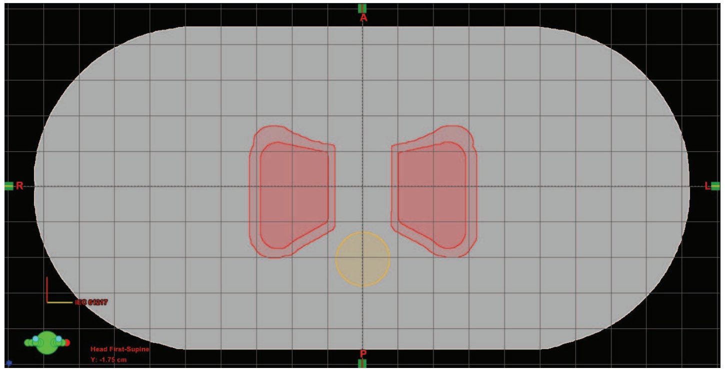
A
R
L
P
IEC 61217
Head First-Supine
Y: -1.75 cm

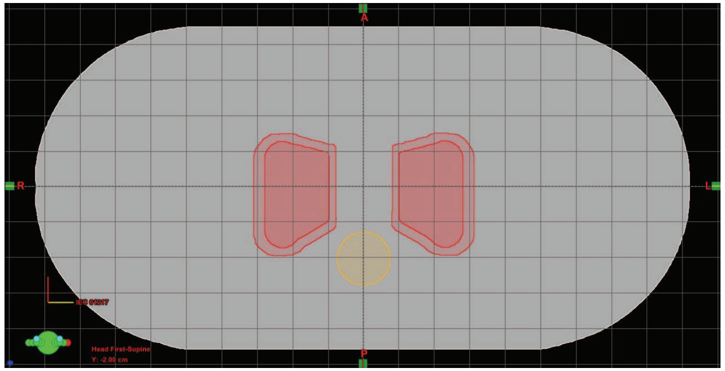
A
R
L
P
IEC 61217
Head First-Supine
Y: -2.00 cm

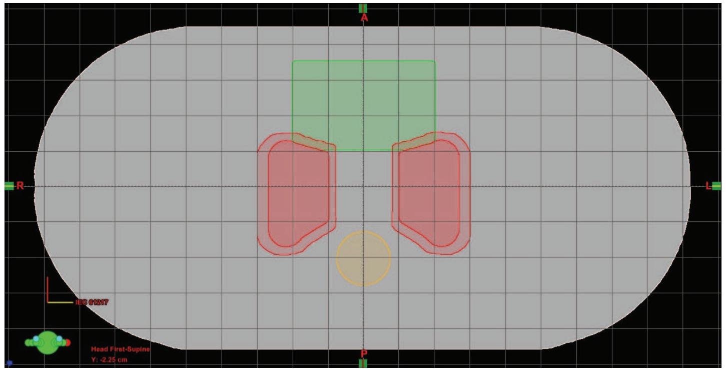
A
R
L
P
IEC 61217
Head First-Supine
Y: -2.25 cm

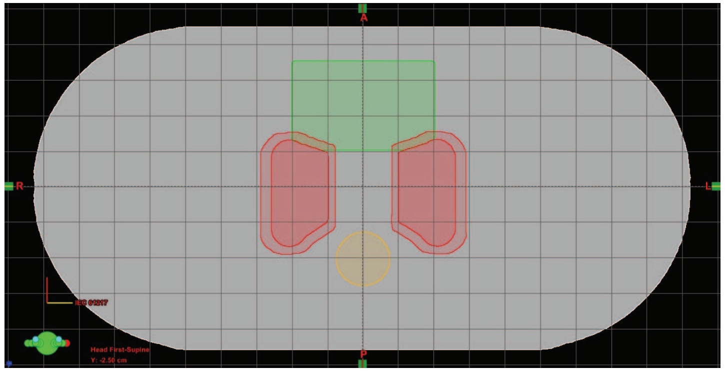
A
R
L
P
IEC 61217
Head First-Supine
Y: -2.50 cm

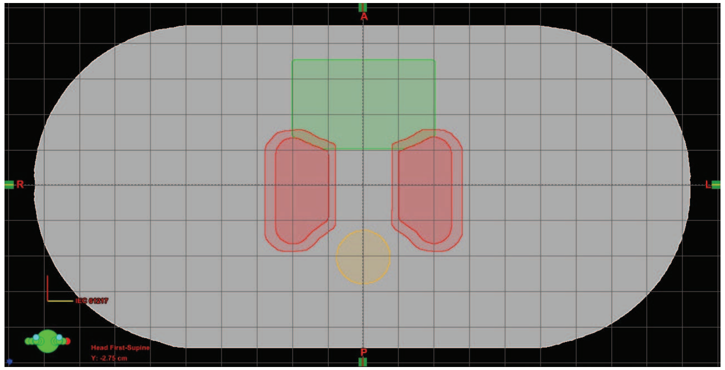
A
R
L
P
IEC 61217
Head First-Supine
Y: -2.75 cm

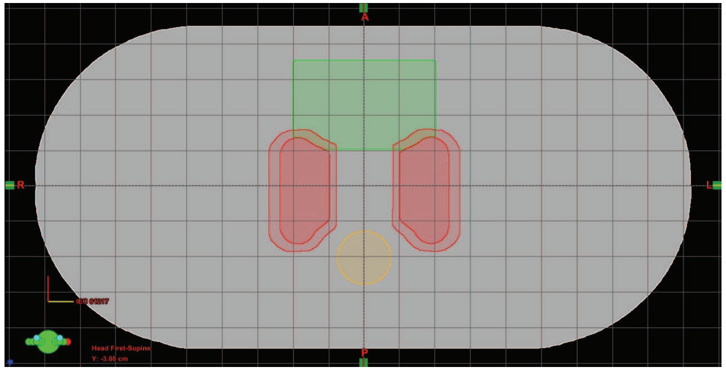
A
R
L
P
IEC 61217
Head First-Supine
Y: -3.00 cm

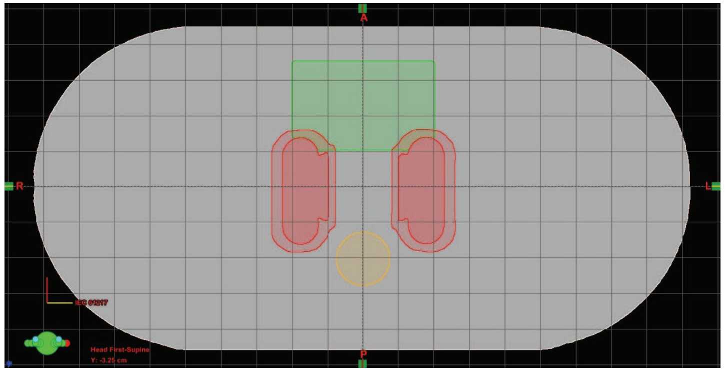
A
R
L
P
IEC 61217
Head First-Supine
Y: -3.25 cm

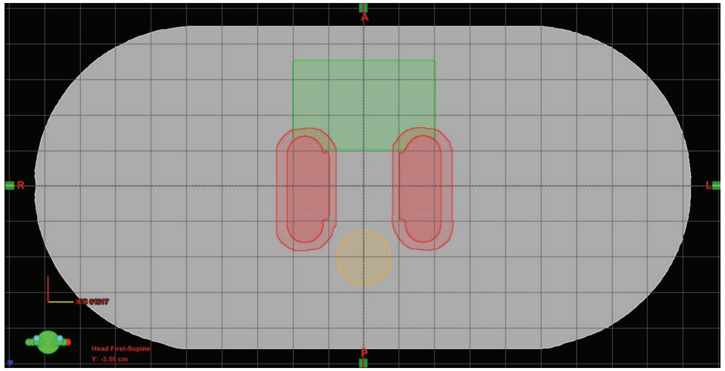
A
R
L
P
IEC 61217
Head First-Supine
Y: -3.50 cm

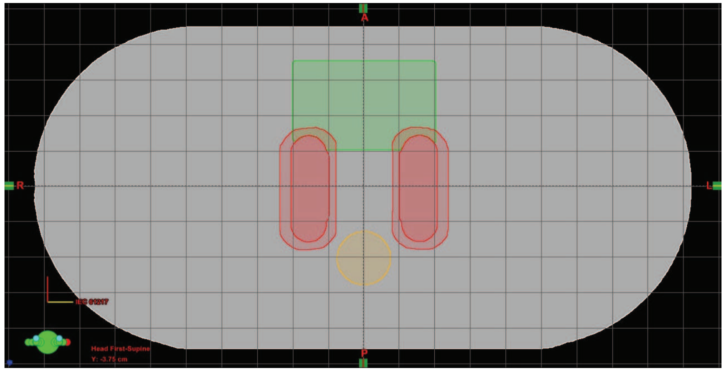
A
R
L
P
IEC 61217
Head First-Supine
Y: -3.75 cm

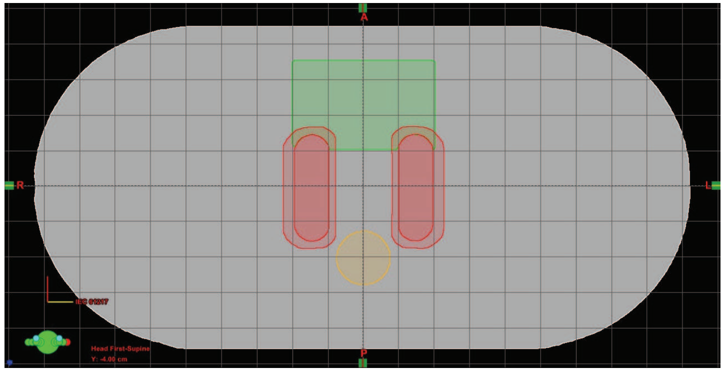
A
R
L
P
IEC 61217
Head First-Supine
Y: -4.00 cm

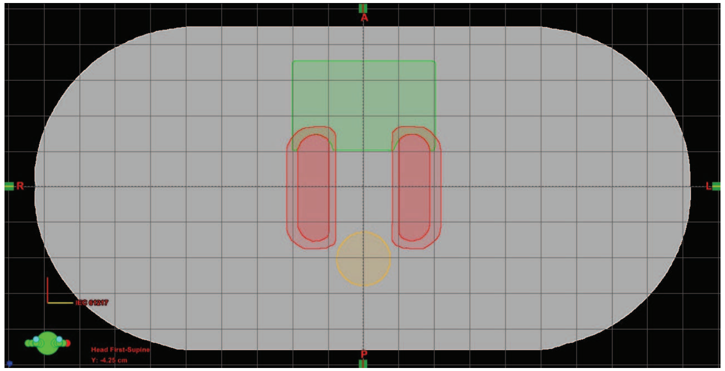
A
R
L
P
IEC 61217
Head First-Supine
Y: -4.25 cm

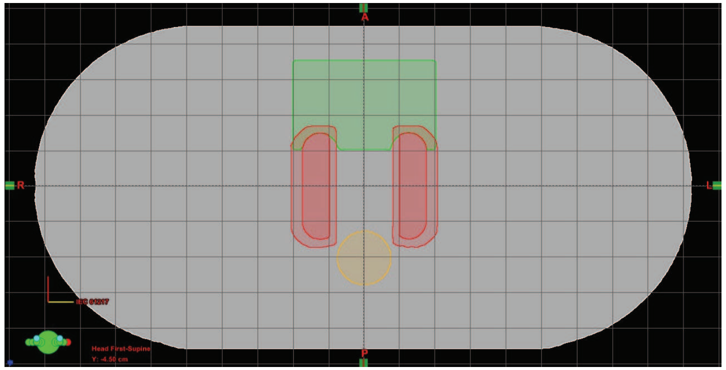
A
R
L
P
IEC 61217
Head First-Supine
Y: -4.50 cm

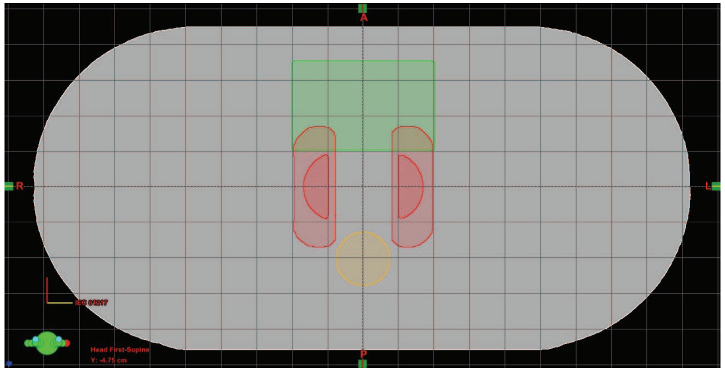
A
R
L
P
IEC 61217
Head First-Supine
Y: -4.75 cm

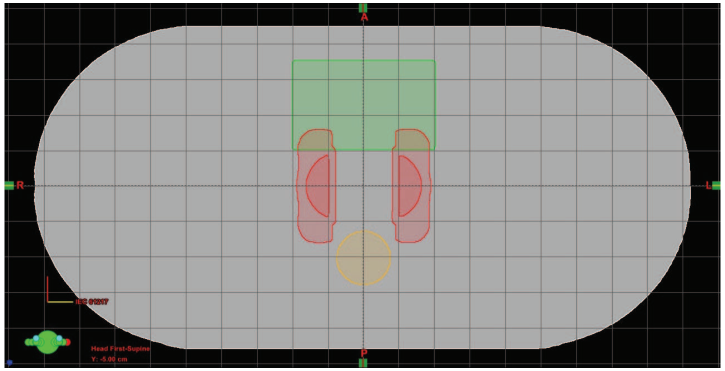
A
R
L
P
IEC 61217
Head First-Supine
Y: -5.00 cm

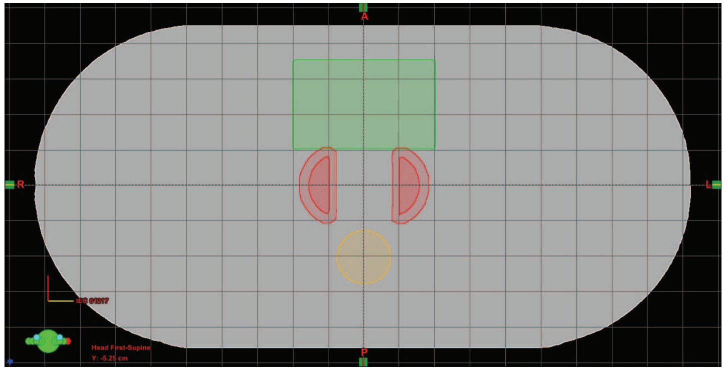
A
R
L
P
IEC 61217
Head First-Supine
Y: -5.25 cm

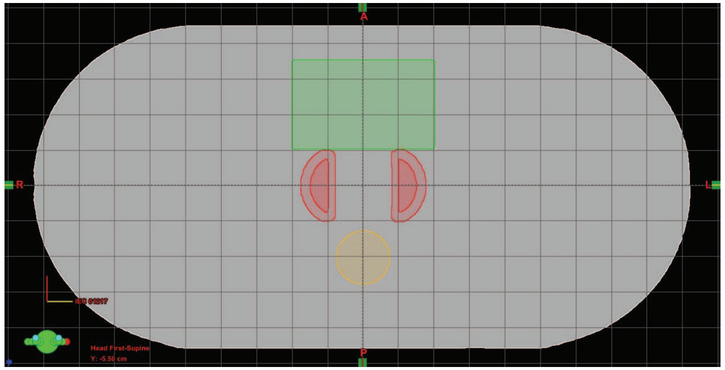
A
R
L
P
IEC 61217
Head First-Supine
Y: -5.50 cm

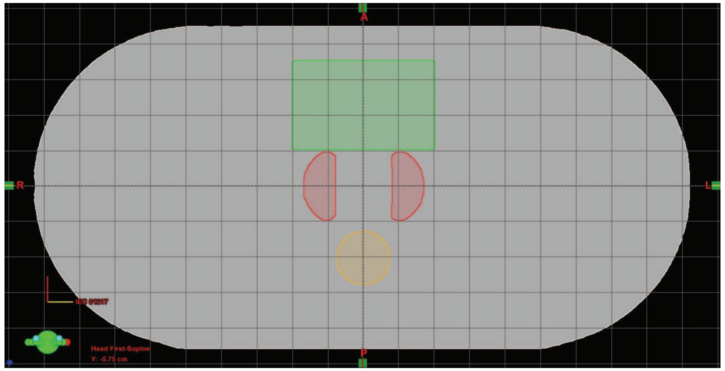
A
R
L
P
IEC 61217
Head First-Supine
Y: -5.75 cm

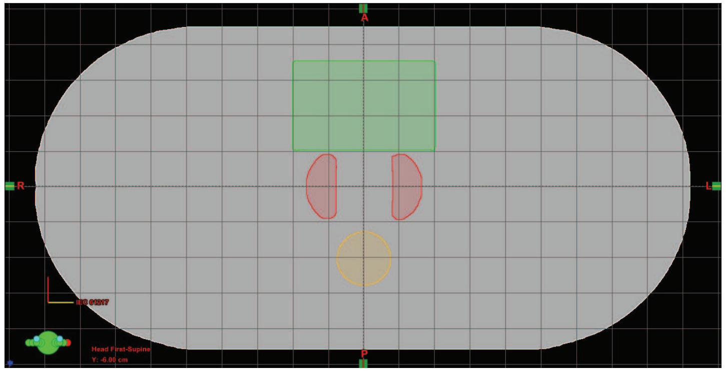
A
R
L
P
IEC 61217
Head First-Supine
Y: -6.00 cm
