# Supplementary material for: Dosimetric comparison of three-dimensional conformal radiotherapy versus volumetric-arc radiotherapy in cervical cancer treatment: applying the central-shielding principle to modern technology
Source: J Radiat Res. 2018 Jul 21;59(5):639–48. doi: 10.1093/jrr/rry054 (PMC6151642; doi:10.1093/jrr/rry054)
Supplement: Supplementary Data [file rry054_supplementary_table_1.pdf]

Supplementary Table 1: Patients' characteristic (average ± standard deviation).

Height (cm)	156.0 ± 6.9
Body weight (kg)	53.8 ± 8.5
BMI	22.0 ± 2.8
FIGO Stage (IB1/IB2/IIB1/IIB2)	4/1/3/2
Tumor size (mm)	
Right-left	37 ± 12
Anterior-posterior	30 ± 10
